# NetNorM: Capturing cancer-relevant information in somatic exome mutation data with gene networks for cancer stratification and prognosis

**DOI:** 10.1371/journal.pcbi.1005573

**Published:** 2017-06-26

**Authors:** Marine Le Morvan, Andrei Zinovyev, Jean-Philippe Vert

**Affiliations:** 1 MINES ParisTech, PSL Research University, CBIO-Centre for Computational Biology, 75006 Paris, France; 2 Institut Curie, 75248 Paris Cedex 5, France; 3 INSERM, U900, 75248 Paris Cedex 5, France; 4 Department of Mathematics and Applications, Ecole normale supérieure, CNRS, PSL Research University, 75005 Paris, France; University of Cambridge, UNITED KINGDOM

## Abstract

Genome-wide somatic mutation profiles of tumours can now be assessed efficiently and promise to move precision medicine forward. Statistical analysis of mutation profiles is however challenging due to the low frequency of most mutations, the varying mutation rates across tumours, and the presence of a majority of passenger events that hide the contribution of driver events. Here we propose a method, NetNorM, to represent whole-exome somatic mutation data in a form that enhances cancer-relevant information using a gene network as background knowledge. We evaluate its relevance for two tasks: survival prediction and unsupervised patient stratification. Using data from 8 cancer types from The Cancer Genome Atlas (TCGA), we show that it improves over the raw binary mutation data and network diffusion for these two tasks. In doing so, we also provide a thorough assessment of somatic mutations prognostic power which has been overlooked by previous studies because of the sparse and binary nature of mutations.

## Author summary

The transition from a normal cell to a cancer cell is driven by genetic alterations, such as mutations, that induce uncontrolled cell proliferation. With the advent of next-generation sequencing technologies (NGS) in the last decade, thousands of tumours have been sequenced and their mutation profiles determined. However, the statistical analysis of these mutation profiles remains challenging. Indeed, two patients usually do not share the same set of mutations and can even have none in common. Moreover, it is difficult to distinguish the few disease-causing mutations from the dozens, often hundreds of mutations observed in a tumour. To alleviate these challenges, it has been proposed to use gene-gene interaction networks as prior knowledge, with the idea that if a gene is mutated and non-functional, then its interacting neighbours might not be able to fulfil their function as well. Here we propose NetNorM, a method that transforms mutation data using gene networks so as to make mutation profiles more amenable to statistical learning. We show that NetNorM significantly improves the prognostic power of mutation data compared to previous approaches, and allows defining meaningful groups of patients based on their mutation profiles.

## Introduction

Tumourigenesis and cancer growth involve somatic mutations which appear and accumulate during cancer progression. These mutations impair the normal behaviour of various cancer genes, and give cancer cells an often devastating advantage to proliferate over normal cells [1–3]. Systematically assessing and monitoring somatic mutations in cancer therefore offers the opportunity not only to better understand the biological processes involved in the disease, but also to help rationalise patient treatment in a clinical setting. Rationalising treatment involves finely characterising the genomic abnormalities of each given patient to discover which may be treatable by a targeted therapeutic agent, as well as improving prognosis using molecular information [4–6]. The development of fast and cost-effective technologies for high-throughput sequencing in the last decade has triggered the launch of numerous data collection projects such as The Cancer Genome Atlas (TCGA) [7] or the International Cancer Genome Consortium (ICGC) [8], aiming at characterising at the molecular level, including genome-wide or exome-wide somatic mutations, thousands of cancer samples of multiple origins. By systematically comparing the molecular portraits of the resulting cohorts, one might expect to be able to detect frequently mutated genes or groups of genes, and find associations between particular mutations and cancer phenotypes, response to treatment, or survival [9–12].

The analysis of somatic mutation profiles is however challenging for multiple reasons. First, most somatic mutations detected by systematic sequencing are likely to be irrelevant for biological or clinical applications. This is due to the fact that only a few driver mutations are required to confer a growth advantage to the cancer cell, and therefore most somatic mutations are likely to be passenger mutations which do not contribute to the cancer phenotype [3, 13]. Second, sequencing efforts have shown that while a few genes are frequently mutated, the vast majority of genes are mutated in only a handful of patients [14, 15]. As a result, the mutation profiles of two tumours often only share a few if any genes in common. Third, even if originating from the same tissue, tumours may exhibit widely varying mutation rates. The overall mutational burden of a tumour constitute a strong and informative signal [16–18] but can however complicate the retrieval of more subtle signals. Combined with the inherent high dimensionality of somatic mutation datasets, this makes any statistical analysis of cohorts of whole-exome somatic mutation profiles extremely challenging.

In order to make somatic mutation profiles more amenable to statistical analysis, several studies have used gene networks as prior knowledge [19, 20]. Considering genes in the context of networks instead of analysing them independently allows sharing mutation information among neighbouring genes and identifying disruptions at the level of pathways or protein complexes instead of single genes. A popular method to leverage this prior knowledge consists in using a diffusion process on the gene network. This technique first appeared for the analysis of gene expression and GWAS data [21–25], and has more recently been used for mutation profiles [26–31]. Network diffusion processes allow smoothing binary vectors of somatic gene mutations into non-negative real-valued vectors of mutational statuses, where the mutational status of a gene increases when it is close to mutated genes in the network. This approach led to state-of-the-art methods for the discovery of driver pathways or complexes [30] and for the stratification of patients into clinically relevant subtypes [31] using whole-exome mutation profiles.

In this work we propose NetNorM, a new method to enhance mutation data with gene networks. NetNorM transforms a patient’s binary mutation profile by either removing mutations or creating “proxy” mutations based on the gene network topology, until all patients reach a consensus number of mutations. The resulting mutation matrix is binary like the initial one, nonetheless we establish that it encodes new information reflecting both local network neighbourhood mutational burdens and the overall tumour mutational burden.

We evaluate the relevance of NetNorM on two tasks: survival prediction and patient stratification from exome somatic mutation profiles. In doing so, we also provide a thorough assessment of somatic mutations prognostic power which has been overlooked by previous studies because of the sparse and binary nature of mutations [32]. We show that NetNorM produces state-of-the-art results for these two tasks compared to the raw binary mutation data and to network diffusion-based methods. By comparing results obtained with real versus randomised networks, we further show that the increase in relevance is actually partly driven by the gene’s network prior knowledge. However, we observe that considering interactions between mutated genes and their network neighbours only is enough do achieve state-of-the-art results, thereby shedding light on which are the network features that are the most informative.

## Results

### Overview of NetNorM

**Fig 1:**
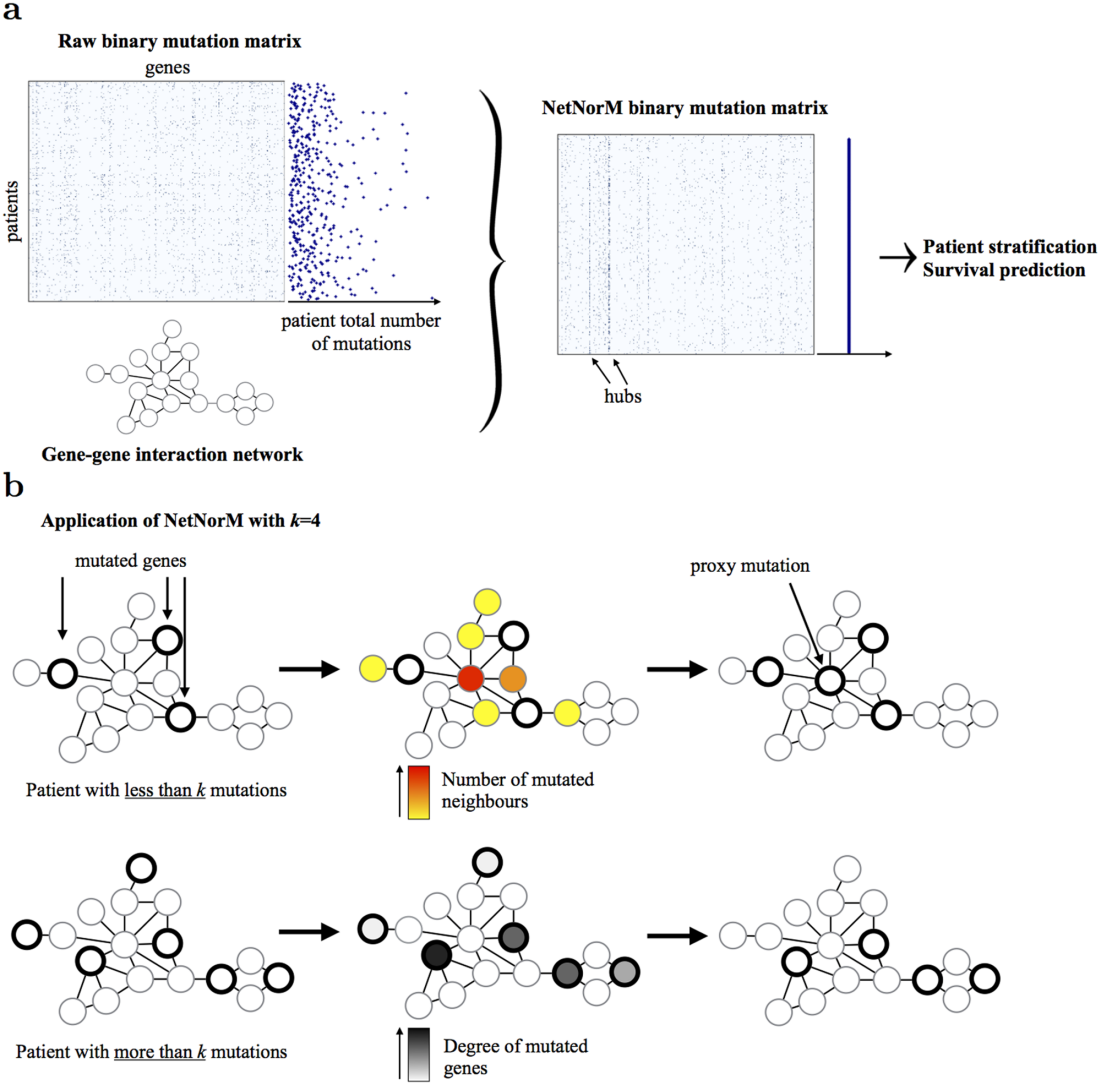
Overview of NetNorM. (a) Using a gene network as background knowledge (lower left), NetNorM normalises each mutation profile in a collection of somatic mutation profiles (upper left) into a new, binary representation (right) which encodes additional information relative to tumours’ overall mutational burden and hubs’ neighbourhood mutational burden. This new representation allows performing patient stratification with unsupervised clustering techniques, or survival analysis. (b) NetNorM normalises every patient mutation profile to *k* mutations. Patients with less than *k* mutations get ‘proxy’ mutations in their genes with the highest number of mutated neighbours until they reach *k* mutations. Patients with more than *k* mutations have mutations ‘removed’ in their genes with lowest degree until they reach *k* mutations.

NetNorM takes as input an undirected gene network and raw exome somatic mutation profiles and outputs a new representation of mutation profiles which allows better survival prediction and patient stratification from mutations (Fig 1). Here and in what follows, the “raw” mutation profiles refer to the binary patients times genes matrix where 1s indicate non-silent somatic point mutations or indels in a patient-gene pair and 0s indicate the absence of such mutations. The new representation of mutation profiles computed with NetNorM also takes the form of a binary patients times genes mutation matrix, yet with new properties. While different tumours usually harbour different number of mutations, with NetNorM all patient mutation profiles are normalised to the same number *k* of genes marked as mutated. The final number of mutations *k* is the only parameter of NetNorM, which can be adjusted by various heuristics, such as the median number of mutations in the original profiles, or optimised by cross-validation for a given task such as survival prediction. In order to represent each tumour by *k* mutations, NetNorM adds “missing” mutations to samples with less than *k* mutations, and removes “non-essential” mutations from samples with more than *k* mutations. The “missing” mutations added to a sample with few mutations are the non-mutated genes with the largest number of mutated neighbours in the gene network, while the “non-essential” mutations removed from samples with many mutations are the ones with the smallest degree in the gene network. These choices rely on the simple ideas that, on the one hand, genes with a lot of interacting neighbours mutated might be unable to fulfil their functions and, on the other hand, mutations in genes with a small number of interacting neighbours might have a minor impact compared to mutations in more connected genes.

In this study, we compare NetNorM-processed profiles with the raw mutation data and with profiles processed with network smoothing (NS) [33] (also called network diffusion, or network propagation) followed by quantile normalisation (QN) as implemented in [31]. We refer to this method as NSQN below. Mutation profiles, either raw or processed with NetNorM or NSQN, are restricted to the genes present in the network used. While both NetNorM and NSQN leverage gene network prior knowledge to enhance mutation data, the two methods have fundamental differences. First, NetNorM leverages information about first neighbours in the network only while NSQN spreads mutation information at a more global scale on the gene network. Second, with NetNorM the normalised profiles all have the same value distribution by construction, since they are all binary vectors with *k* ones, removing the need for further quantile normalisation which, as we discuss below, is critical for NSQN.

### NetNorM provides state-of-the-art prognosis for patient survival based on mutation profiles

To assess the relevance of NetNorM, we first explore the capacity of somatic mutations to predict patient survival. We collected a total of 3,278 full-exome mutation profiles of 8 cancer types from the TCGA portal (Table 1), censored survival information and clinical data. In parallel we retrieved a gene network to be used as background information for NSQN and NetNorM: Pathway Commons, which integrates a number of pathway and molecular interaction databases [34]. For each cancer type, we use these data to assess how well survival can be predicted from somatic mutations. For that purpose, we perform survival prediction with a sparse survival SVM (see Methods) using either the raw mutation profiles or the profiles processed with NSQN or NetNorM, respectively, and assess their performance by cross-validation using the concordance index (CI) on the test sets as performance metric.

**Table 1:** Summary of the full exome mutation profiles used in this study. We analysed a total of 3,278 samples from 8 cancer types, downloaded from the TCGA portal.

Cancer type	Patients	Genes	Deaths	Download date
LUAD (Lung adenocarcinoma)	430	20 596	110	6/22/2015
SKCM (Skin cutaneous melanoma)	307	17 461	129	11/18/2015
GBM (Glioblastoma multiform)	265	14 748	195	11/18/2015
BRCA (Breast invasive carcinoma)	945	16 806	97	11/25/2015
KIRC (Kidney renal clear cell carcinoma)	411	10 608	136	11/25/2015
HNSC (Head and Neck squamous cell carcinoma)	388	17 022	140	11/25/2015
LUSC (Lung squamous cell carcinoma)	169	13 589	70	11/25/2015
OV (Ovarian serous cystadenocarcinoma)	363	10 192	172	11/24/2014

**Fig 2:**
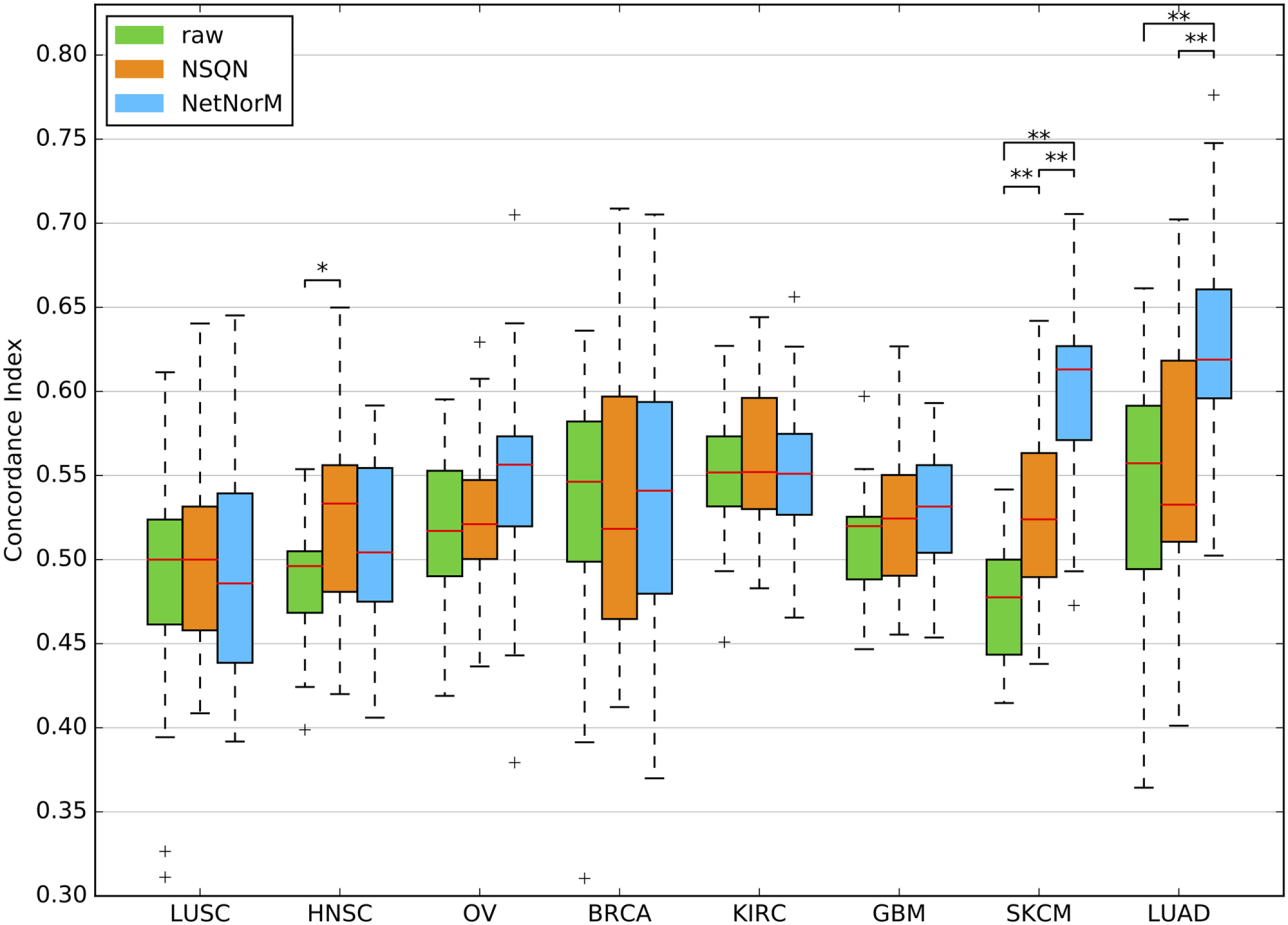
Comparison of the survival predictive power of the raw mutation data, NSQN and NetNorM (with Pathway Commons as gene network) for 8 cancer types. For each cancer type, samples were split 20 times in training and test sets (4 times 5-fold cross-validation). Each time a sparse survival SVM was trained on the training set and the test set was used for performance evaluation. The presence of asterisks indicate when the test CI is significantly different between 2 conditions (Wilcoxon signed-rank test, *P* < 5 × 10^−2^ (*) or *P* < 1 × 10^−2^ (**)).

Fig 2 summarises the survival prediction performances for the 8 cancer types, when the sparse survival SVM is fed with the raw mutation profile, or with the mutation profiles modified by NSQN or NetNorM using Pathway Common as gene network. For two cancers (LUSC, HNSC), none of the methods manages to outperform a random prediction, questioning the relevance of the mutation information in this context. For OV, BRCA, KIRC and GBM, all three methods are significantly better than random, although the estimated CI remains below 0.56, and we again observe no significant difference between the raw data and the data transformed by NSQN or NetNorM. Finally, the last two cases, SKCM and LUAD, are the only ones for which we reach a median CI above 0.6. In both cases, processing the mutation data with NetNorM significantly improves performances compared to using the raw data or profiles processed with NSQN. More precisely, for LUAD the median CI increases from 0.56 for the raw data and 0.53 for NSQN to 0.62 for NetNorM. In the case of SKCM, the median CI increases from 0.48 for the raw data to 0.52 for NSQN, and to 0.61 for NetNorM. For SKCM, both NetNorM and NSQN are significantly better than the raw data (*P* < 0.01).

In our experiments, silent mutations are systematically filtered out. To evaluate whether this preprocessing step is actually detrimental or beneficial for the survival prediction task, we performed further experiments where silent mutations are not filtered out (S1 Fig). We find that considering silent mutations does not improve survival prediction performances compared to the case where they are filtered out. In fact, the performance of NetNorM on LUAD is significantly decreased when silent mutations are taken into account.

To assess the influence of the gene network used on the survival prediction performances, we also repeated our experiments with four gene networks instead of Pathway Commons: BioGRID [35], HPRD [36], HumanNet [37] and STRING [38] (S2 Fig). For HumanNet and STRING, only the 10% most confident interactions were retained. We observe that no gene network clearly stands out as the best network for all cancers. For two cancers, LUSC and HNSC, performances remain very low, close to a concordance index of 0.5, whatever the method or network used. For three cancers, OV, BRCA and KIRC, NetNorM is the only method to significantly outperform the raw data with at least one network (HumanNet and STRING for OV, HPRD for BRCA, and STRING for KIRC) with a median concordance index above 0.55. For GBM, NSQN is the only method to outperform the raw data (with HumanNet and STRING) with a median concordance index above 0.55. For the two remaining cancers, LUAD and SKCM, the best performances are those obtained with NetNorM using Pathway Commons, with median CI of 0.62 and 0.61 respectively. Across all cancers, methods, and networks combinations, these two cases are the only ones where the median CI obtained exceeds 0.60.

Finally, as mutations in some genes are known to be associated with survival, such as *TP53* in BRCA and HNSC which is associated with worsened survival [39], we evaluate the prediction ability of individual genes’ mutation status. For each cross-validation fold, the gene giving the best concordance index on the training set is selected and its performance evaluated on the test set. We find that for 5 cancers, the performances of individual genes are similar to those of the survival SMV applied to the whole raw mutations datasets (S3 Fig). However for BRCA and HNSC, better survival predictions are obtained using a single gene than the whole raw mutational profiles. Yet these predictions are not better than those obtained with NetNorM. For these two cases, *TP53* is the gene selected in the majority of folds (17/20 for HNSC and 19/20 for BRCA), which is in accordance with existing literature (S1 Table). Lastly, the survival SVM applied to the whole dataset yields significantly better performances than the single gene approach for LUAD. This means that contrary to the BRCA and HNSC cases, the linear combinations of genes which are found for LUAD have a predictive power that generalises well to unseen data.

In summary, these results show that for at least 6 out of 8 cancers investigated, somatic mutation profiles have a prognostic value, and that for two of them (SKCM and LUAD) it is possible to improve the prognostic power of mutations by using gene networks and to reach a CI above 0.6. In both cases, NetNorM is significantly better than NSQN.

### The biological information encoded in the gene network contributes to the prognosis

**Fig 3:**
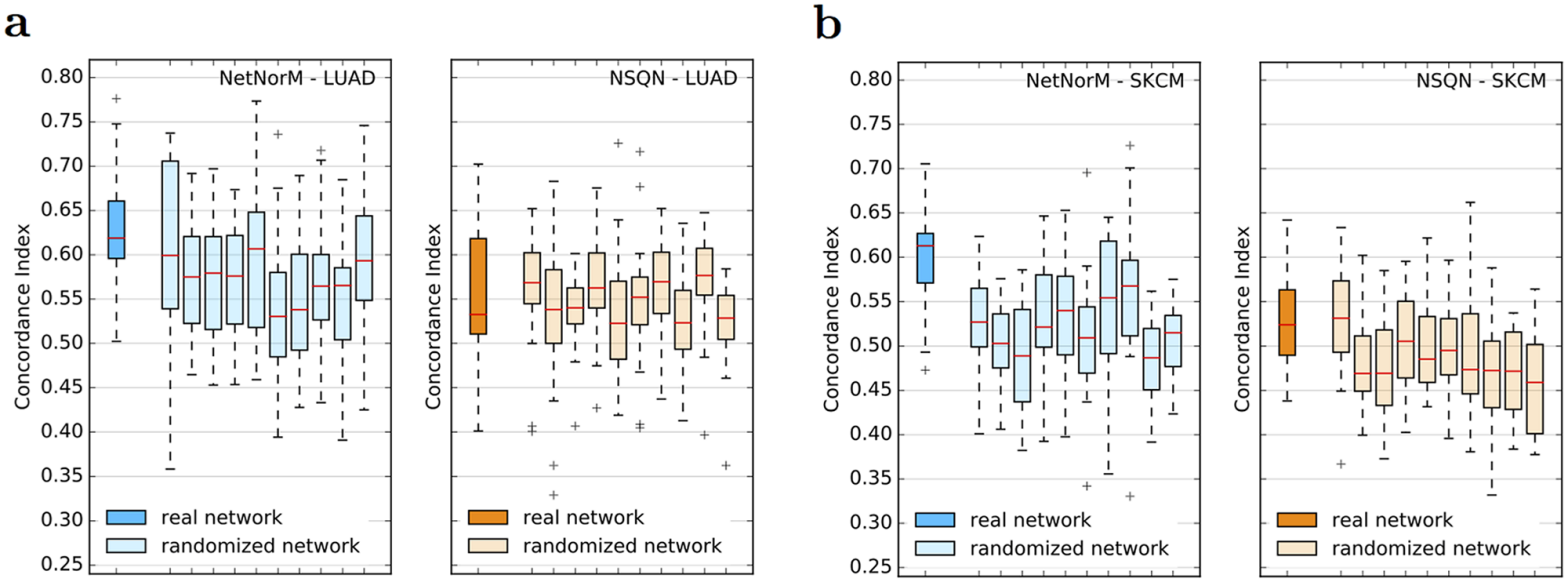
Effect of network randomisation on survival prediction performances. (a-b) Performances obtained for 20 cross-validation folds with Pathway Commons (real network) and 10 randomised versions of Pathway Commons (randomised network) with NetNorM (left) and NSQN (right) for LUAD (a) and SKCM (b).

To test whether the biological information contained in the gene network plays a role in the improvement of survival predictions for LUAD and SKCM, we evaluate again NetNorM and NSQN using 10 different randomised versions of Pathway Commons for these two cancers. Random networks were obtained by shuffling the nodes’ labels of the real network while keeping the structure unchanged. The results, shown on Fig 3, demonstrate that NetNorM performs significantly better with a real network. More precisely, the real network significantly outperforms all random networks for SKCM and 8 out of 10 random networks for LUAD (Wilcoxon signed-rank test with correction for multiple hypothesis testing, FDR ≤ 5%). NSQN also performs significantly better with a real network for SKCM (7 out of 10 cases) but not for LUAD (0 out of 10 cases). This last observation is not surprising since NSQN does not improve over the raw data for LUAD, which suggests that the method may have failed to leverage network information in this case. In summary, these results indicate that the improvements obtained with NetNorM and NSQN compared to the raw data do rely on biological information encoded in the network.

### Analysis of predictive genes

**Fig 4:**
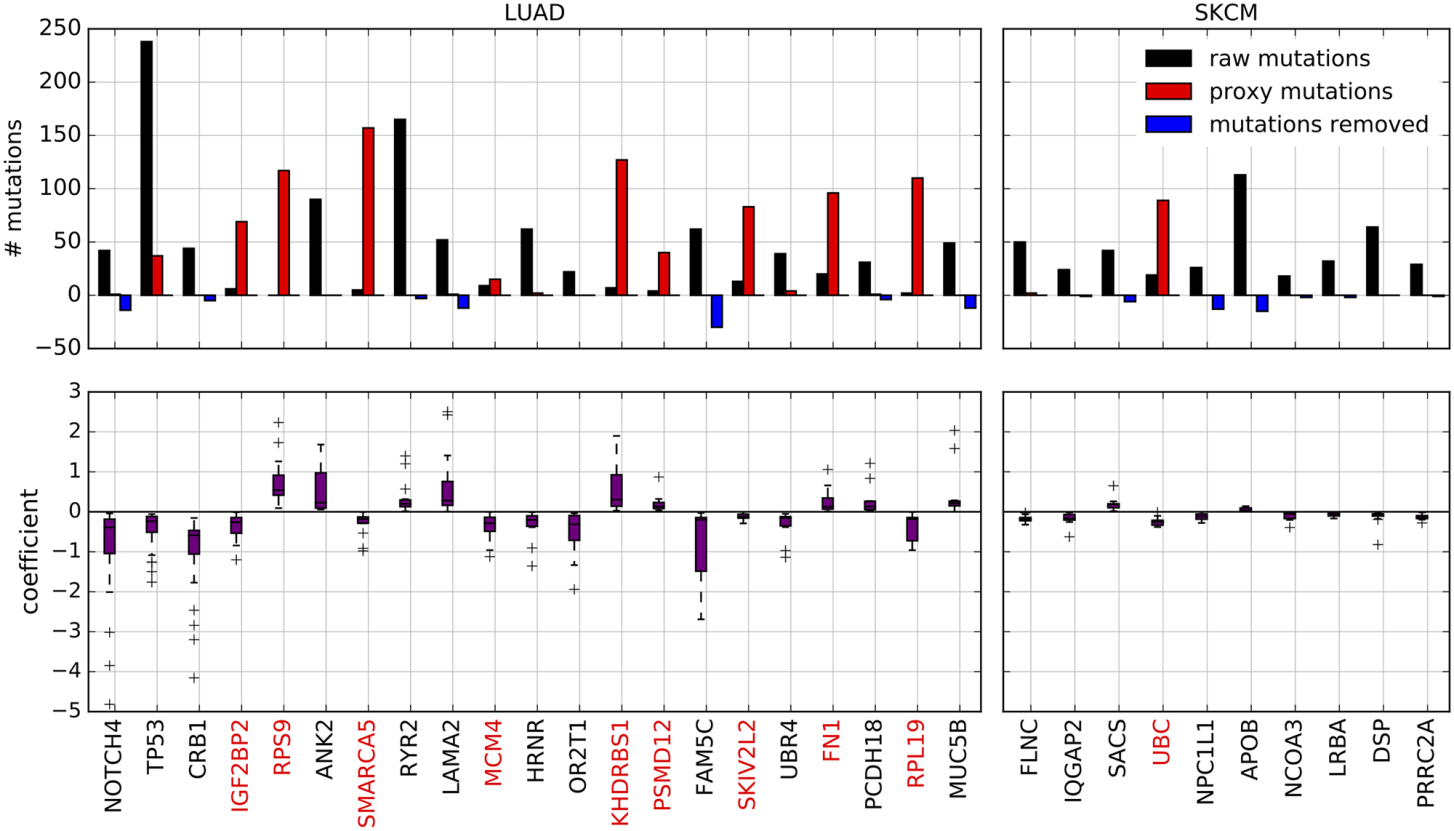
Genes frequently selected in the survival prediction model for LUAD (left) and SKCM (right) learned using the NetNorM representation of mutations with Pathway Commons as gene network. The genes reported are those that were selected at least 10 times in 20 cross-validation folds. For each cancer, genes are ordered from the most frequently selected (left) to the least frequently selected (right). The top panel reports the number of raw mutations in the selected genes (black), as well as the number of “proxy” mutations (red) and the number of mutations removed (blue) after application of NetNorM. The bottom panel reports the coefficients of a gene in the survival SVM model across the cross-validation folds where this gene was selected. Gene names marked in red indicate proxy genes.

In order to shed light on the reasons why NetNorM outperforms the raw data and NSQN on survival prediction for SKCM and LUAD, we now analyse more finely the normalisation carried out by NetNorM on the mutation profiles, and why they lead to better prognostic models. For that purpose, we focus on the genes that are selected at least 50% of the times by the sparse survival SVM during the 20 different train/test splits of cross-validation, after NetNorM normalisation. This leads to 21 frequently selected genes for LUAD and 10 for SKCM (Fig 4). Remembering that NetNorM either removes mutated genes for patients with many mutations, or adds proxy mutations for patients with few mutations, we can assess for each frequently selected gene whether it tends to exhibit proxy mutations or whether it tends to be actually mutated in the tumour. This is done by comparing how frequently it is marked as mutated on the raw data and after NetNorM normalisation (Fig 4, top plot). For both cancers, we observe two clearly distinct groups of frequently selected genes: those that concentrate proxy mutations (which we will call *proxy genes*, in red in Fig 4), and those to which NetNorM brings only few modifications compared to the raw data, meaning they are usually actually mutated in the tumours (in black in Fig 4).

#### Genes with few modifications imputed by NetNorM

In the case of LUAD, 12 out of the 21 selected genes are non-proxy genes, meaning they tend to be really mutated when they are marked as mutated after NetNorM normalisation. Interestingly, mutations in 5 of these genes are predictive of an increased survival time (corresponding to a positive coefficient in the sparse survival SVM) while mutations in the remaining 7 genes are predictive of a decreased survival time (corresponding to a negative coefficient) (Fig 4, bottom plot). The three most important predictors according to their frequency of selection include *NOTCH4*, *TP53* and *CRB1* (selected in all of the 20 folds) and are all predictive of a decreased survival time. *TP53* is a well-known cancer gene and has been reported as significantly mutated in LUAD [40, 41]. *NOTCH4* is part of the *NOTCH* signalling pathway which has been widely implicated in cancer and shown to act as both oncogene or tumour suppressor depending on the context [42]. Finally, *CRB1* is known to localise at tight junctions but little is known about its role in carcinogenesis [43]. Among the remaining genes, *LAMA2* (selected in 16 out of 20 folds) has been detected as a driver gene in head and neck squamous cell carcinoma and *PCDH18* (selected in 11 out of 20 folds) has been detected as a driver in bladder carcinoma, cutaneous melanoma and in a pan-cancer analysis setting [44]. In the case of SKCM, 9 out of the 10 selected genes are genes with few modifications. This includes 7 genes whose mutations are predictive of a decreased survival time (*FLNC*, *IQGAP2*, *NPC1L1*, *NCOA3*, *LRBA*, *DSP*, *PRRC2A*), and 2 whose mutations are predictive of an increased survival time (*SACS* and *APOB*). Among these genes, *NCOA3* (also known as *AIB1* or *SRC3*) is an important oncogene in breast cancer [45, 46]. Its role in other cancers is unclear however it has been shown that overexpression of *NCOA3* is a marker of melanoma outcome [47]. *LRBA* interacts with multiple important signal transduction pathways including *EGFR* and its deregulation in several cancer types has been shown to facilitate cancer cell growth [48]. Moreover *LRBA* expression has been indicated as a clinical outcome predictor in breast cancer [49]. *Filamin C* (*FLNC*, selected in all of the 20 folds) is a large actin-cross-linking protein which has been shown to inhibit proliferation and metastasis in gastric and prostate cancer cell lines [50]. *Desmoplakin* (*DSP*) is required for functional desmosomal adhesion which has been linked to cancer cells development and progression in several cancers [51, 52]. Moreover *IQGAP2* has been identified as a tumour suppressor gene in hepatocellular carcinoma, gastric and prostate cancers [53].

#### Proxy genes

**Fig 5:**
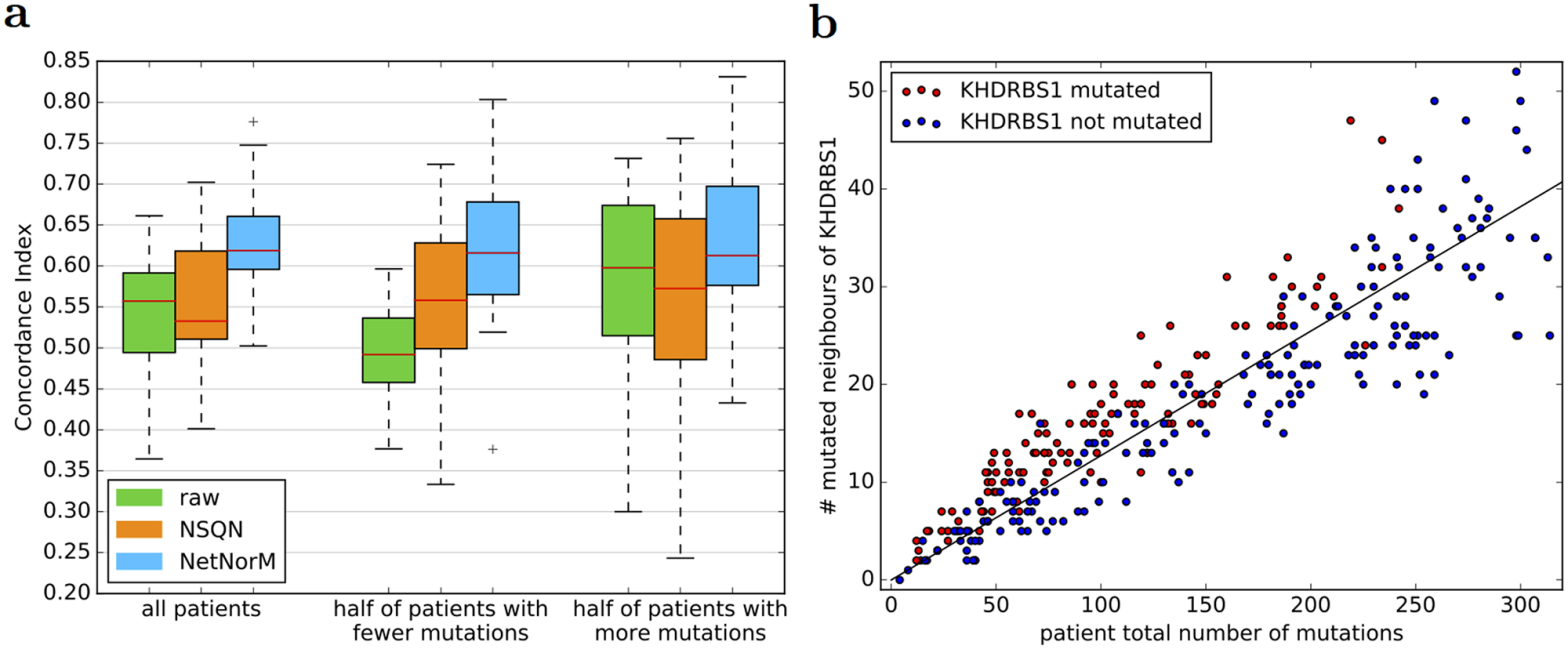
Analysis of predictive genes. (a) Comparison of survival prediction performances according to patients’ mutational burden for LUAD. Three different representations of the mutations are used to perform survival prediction using a ranking SVM: *raw* (the raw binary mutation data), *NSQN* (network smoothing with quantile normalisation) and *NetNorM*. Performances for half of the patients with fewer (resp. more) mutations are derived from the predictions made using the whole dataset. (b) Scatter plot of the total number of mutations in a patient of the LUAD cohort (x-axis) against the number of mutated neighbours of *KHDRBS1* in a patient (y-axis). Only patients with less than *k*_*med*_ = 295 mutations are shown, where *k*_*med*_ is the median value of *k* learned across cross-validation folds. Red (resp. blue) indicate patients mutated (resp. non mutated) in *KHDRBS1* after processing with NetNorM using *k* = *k*_*med*_. The black line was fit by linear regression and by definition indicates the expected number of mutated neighbours of *KHDRBS1* given the mutational burden of a patient.

In addition to somatically mutated genes, several proxy genes, mutated by the NetNorM procedure, are often selected by the survival model. The proxy genes for LUAD are *IGF2BP2*, *RPS9*, *SMARCA5*, *MCM4*, *KHDRBS1*, *PSMD12*, *SKIV2L2*, *FN1*, *RPL19* and for SKCM *UBC* is the only one. These genes are among the biggest hubs in the network. This is expected as proxy mutations are imputed in genes with a lot of mutated neighbours, which is more likely to occur for genes that simply have a lot of neighbours. The fact that these proxy genes were selected in the survival models means that they have some prognostic power. In particular for LUAD, the better prediction performances achieved by NetNorM compared to the raw data is largely explained by better predictions made for the half of patients with fewer mutations, and therefore by the proxy mutations that were created in these patients (Fig 5a).

The prognostic power of proxy genes in NetNorM comes from at least two types of information they capture. The first type of information captured by proxy mutations is the total number of mutations in a patient. Patients harbouring proxy mutations are significantly less mutated than those without proxy mutations (Welsh t-test, *P* ≤ 1 × 10^−2^) in a given proxy gene. This results from the fact that patients with few mutations receive as many proxy mutations as needed to reach the target number of mutations *k*, and therefore proxy mutations have a higher probability to be set in patients with few mutations. The fact that NetNorM creates proxies for the total number of mutations raises the question of whether or not the total number of mutations can improve survival predictions made using the raw binary mutation profiles. To answer this question, we trained a model to predict survival from the raw binary mutation profiles concatenated with a feature, scaled to unit variance, which records the total number of mutations in each patient (S4 Fig). According to our results, taking into account such a feature does not improve survival prediction performances compared to using the raw data alone. We therefore tested another feature which better mimics the proxies created by NetNorM, which we call ‘proxies’. This feature is equal to the total number of mutations in a patient for patients with less than *k* mutations, and is equal to 0 otherwise. We trained a survival prediction model on the raw data concatenated with the feature ‘proxies’, scaled to unit variance, where *k* is chosen by cross-validation. Interestingly, we find that using such a feature allows to significantly improve the results obtained for OV, KIRC and LUAD compared to the raw data alone. In particular, the performances obtained for LUAD are on par with those obtained with NetNorM, suggesting that the feature ‘proxies’ summarises well the information leveraged by NetNorM. However this is not the case for SKCM since considering the feature ‘proxies’ does not improve over using the raw data alone. We draw two conclusions from these observations: first, NetNorM creates relevant proxies for the total number of mutations which, in combination with the binary mutation profiles, have predictive power; second, such proxies do not entirely explain the performances of NetNorM, at least for SKCM.

The second type of information captured by proxy mutations is genes’ neighbourhood mutational burden (NMB). When we look at which patients get mutated in a given gene after NetNorM normalisation (red dots in Fig 5b), we observe that they tend to have more mutations in the neighbours of this gene than what the sole mutational burden would predict (represented by the regression line in Fig 5b). In other words, among the hubs that could get mutated by NetNorM for patients with few mutations, the ones that get mutated tend to be the ones surrounded by more mutations than expected given the mutational burden of the patient. NetNorM thus creates proxy mutations when a gene’s NMB is higher than expected.

Among the proxy genes selected in LUAD (resp. SKCM), *IGF2BP2*, *SMARCA5*, *MCM4*, *PSMD12* and *SKIV2L2* (resp. *UBC*) define groups of patients with significantly different survival outcomes (log-rank test, *P* ≤ 5 × 10^−2^). Given the discussion in the previous paragraph, this may be due to differences in the overall mutational burden between tumours, to differences in NMB for some genes, or to both effects. To clarify the contributions of each effect, we investigate whether such distinct survival outcomes can be obtained with proxies for the total number of mutations only, regardless of NMBs. To this end, we simulate proxy mutations according to a probability depending on patients’ total number of mutations only. By contrast, NetNorM mutates genes according to patients’ total number of mutations and according to genes’ NMB. Then for each gene we compare the survival outcomes of the obtained subgroups (patients which were imputed a proxy mutation versus those that were not) using a log-rank test and examine whether the log-rank statistic is higher with NetNorM than with the simulations (see Methods for more details). We find that all of *IGF2BP2*, *SMARCA5*, *MCM4*, *PSMD12*, *SKIV2L2* and *UBC* produce groups with a significantly higher log-rank statistic with NetNorM than with their simulated counterpart (log-rank test, *P* ≤ 5 × 10^−2^). This clarifies that the prognostic information captured by proxy mutations with NetNorM combines the overall mutational burden of the patient with local mutational burden on the gene network.

### NetNorM enhances clinical data based prognosis

**Fig 6:**
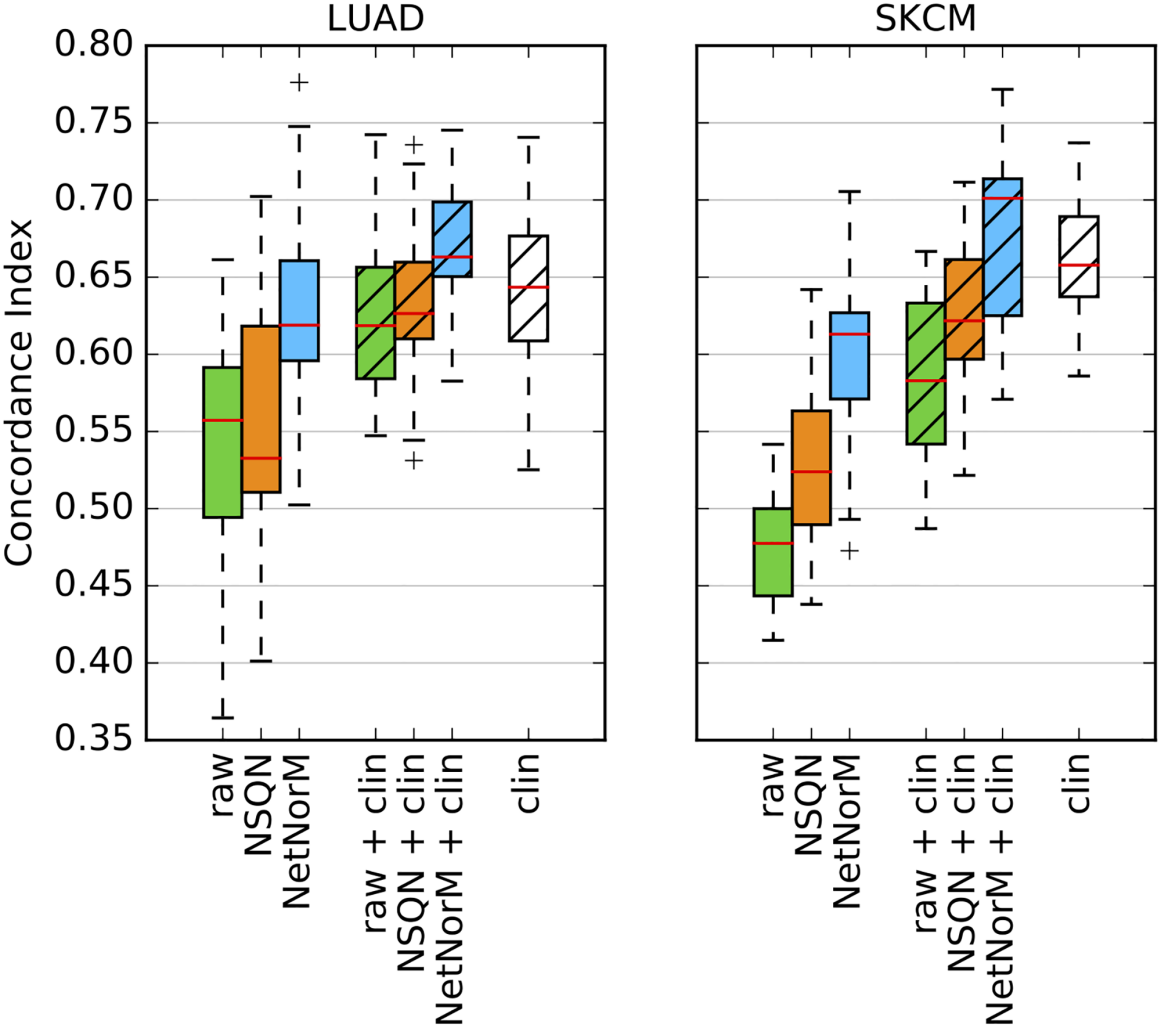
Survival predictive power of mutation data (raw binary mutations, mutations preprocessed with NSQN or NetNorM with Pathway Commons), clinical data, and the combination of both for LUAD and SKCM. The combination of both data types was made by averaging the predictions obtained with each data type separately. For both cancers, samples were split 20 times in training and test sets (4 times 5-fold cross-validation). Each time a sparse survival SVM was trained on the training set and the test set was used for performance evaluation.

We assess whether the combination of both mutations and clinical features can improve performances for LUAD and SKCM compared to using clinical data alone. For this purpose, two sparse survival SVM models are trained independently: one on the raw mutation data or mutations preprocessed with NSQN or NetNorM and one on the clinical data. Then the survival predictions from both models are simply averaged (after being standardised to unit variance). The resulting predictions are again evaluated in a 4 times 5 folds cross-validation setting. First, the results show that mutations preprocessed with NetNorM and the clinical data yield similar performances (*P* = 0.52, Wilcoxon signed rank test) for LUAD while the clinical data performs significantly better than NetNorM in the case of SKCM (*P* ≤ 1 × 10^−2^) (Fig 6). Moreover, we observe that combining mutations preprocessed with NetNorM with clinical features allows improving survival predictions compared to the clinical data alone for both LUAD (*P* = 4.8 × 10^−2^) and SKCM (*P* = 5.7 × 10^−2^). More precisely, the median CI increases from 0.64 with the clinical data to 0.66 with the combination of NetNorM and the clinical data for LUAD and from 0.66 to 0.70 in the case of SKCM. We also tried to concatenate the mutation profiles with the clinical data before training a unique model and observed that it did not improve the results compared to the previous strategy (S5 Fig). Overall, these results suggest that mutations could provide useful prognostic information that is complementary to the clinical information available.

### NetNorM allows stable unsupervised stratification of patients with significantly different survival curves

We now assess the possibility to stratify patients into a small number of groups in an unsupervised way, meaning without using survival information, in order to identify distinct subgroups of patients in terms of mutational profiles. For that purpose, we use a standard unsupervised clustering pipeline based on nonnegative matrix factorisation (NMF), and apply it to the different cohorts of patients represented by the raw mutation profiles, or the profiles normalised by NSQN or NetNorM. The hyperparameters *k* (NetNorM) and *α* (NSQN) were set to default values chosen as the median number of mutations in a cohort for *k* and *α* = 0.5 as recommended in [31]. As we have no ground truth regarding “true” groups of patients, we assess the quality of clustering by two factors: (i) the stability of the clusters, assessed by the proportion of ambiguous clustering (PAC) which is the rate of discordant cluster assignments across 1,000 random subsamples of the full cohort; and (ii) the significance of association between clusters and survival.

**Fig 7:**
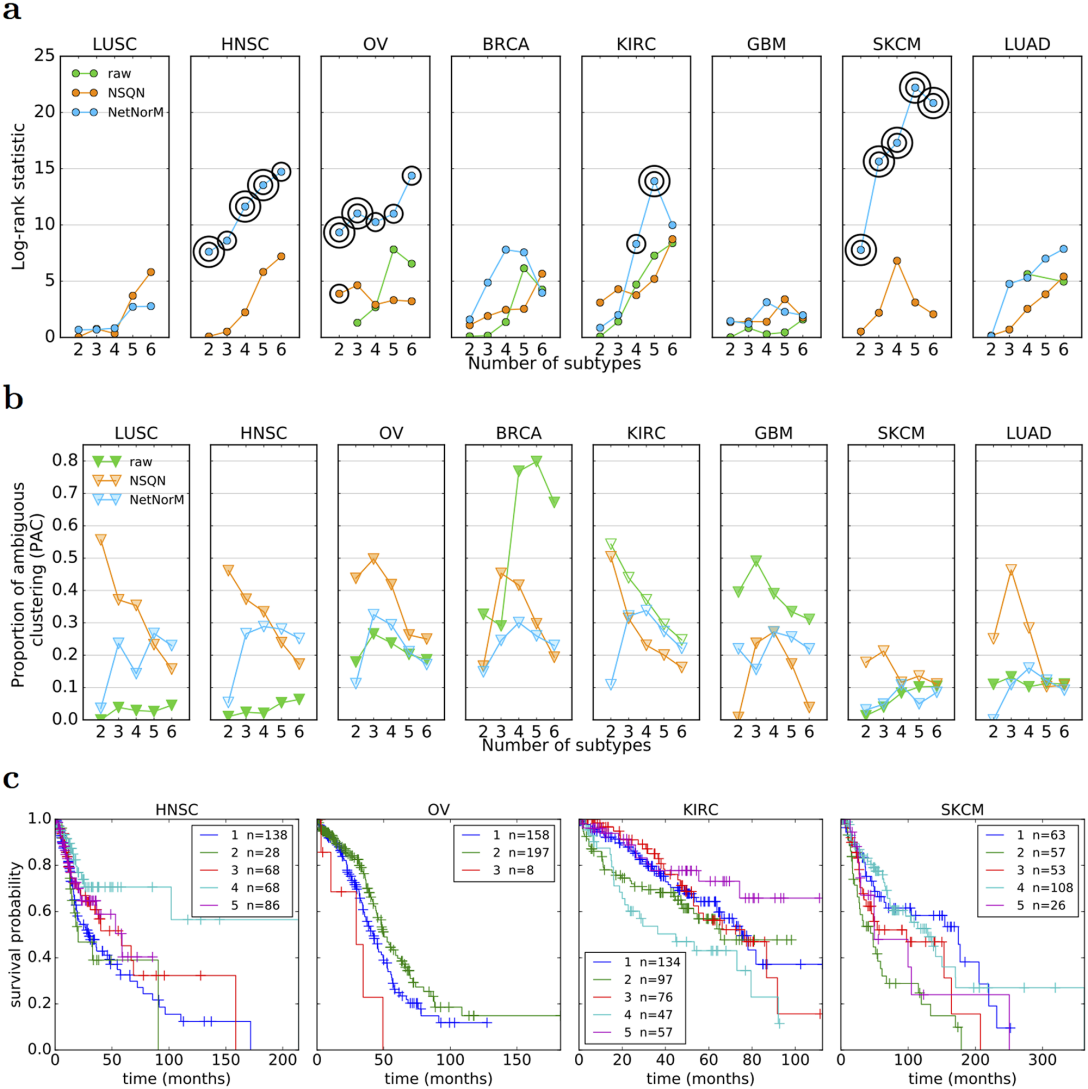
Comparison of patient stratifications obtained with the raw mutation data, NSQN (Pathway Commons) and NetNorM (Pathway Commons) for 8 cancer types. (a) Association of patient subtypes with survival time. One circle indicates *P* ≤ 0.05 and two concentric circles indicate *P* ≤ 0.01 (log-rank test). Cases where clusters were too unbalanced (95% of the patients in one single cluster) are not shown. (b) Evaluation of the clustering stability as measured by the proportion of ambiguous clustering (PAC). The transparency of the triangles indicate the percentage of patients in the largest cluster. The scale ranges from 100% (totally opaque) to $\frac{1}{N}$ % (totally transparent) where *N* is the number of subtypes. Therefore opacity (resp. transparency) indicate unbalanced (resp. balanced) clusters. (c) Kaplan Meir survival curves for NetNorM subtypes with significantly distinct survival outcomes. In the legend are indicated the subtype number followed by the number of patients in the subtype.

With the raw data, NMF tends to stratify patients into very unbalanced subtypes with typically one subtype gathering the majority of patients (Fig 7b). LUSC, HNSC and SKCM are extreme cases where one cluster contains 95% of the patients, whatever the number of clusters. In addition, in cases where the obtained clusters are reasonably balanced as for KIRC, the clustering stability is low. These results are coherent with [31] who highlighted the difficulty to cluster raw mutation profiles. These undesirable behaviours disappear with both NSQN and NetNorM (Fig 7). With NetNorM the obtained clusters are reasonably balanced across all cancers and the clusters are stable (*PAC* ≤ 30%). NSQN also provides stable clusters (*PAC* ≤ 30%) when the number of clusters is set between 4 and 6 however for 2 or 3 clusters the stability is not as good (*PAC* ≤ 50%). To assess the clinical relevance of the obtained subtypes, we test whether they are associated with significantly distinct survival outcomes (Fig 7a). With the raw data, patient stratification is never significantly associated with clinical data. With NetNorM, significant associations of patient subtypes with survival times are achieved for HNSC, OV, KIRC and SKCM (Fig 7c), while with NSQN, a significant association is only achieved for OV. The stratification based on NetNorM remains prognostic beyond clinical data for SKCM (Likelihood ratio test, *P* = 2.4 × 10^−2^ (SKCM, *N* = 5)). It can be surprising at first sight that no signal is recovered for LUAD with NetNorM and for SKCM with NSQN since some signal was observed in the survival prediction setting in these cases. We hypothesized that this could be due to a bad choice of the hyperparameters *k* and *α* for these cancer types. Therefore additional experiments were run for LUAD and SKCM with *k* and *α* set to their values learned by cross-validation for the survival prediction task (S3 Table). This corresponds to *k* = 315 and *α* = 0.6 for LUAD (instead of *k* = 189 and *α* = 0.5 as defaults) and *k* = 140 and *α* = 0.25 for SKCM (instead of *k* = 243 and *α* = 0.5 as defaults). With these new values for the hyperparameters, significant associations with survival are detected for LUAD with NetNorM (for 4, 5 and 6 clusters) and for SKCM with both NetNorM (for any number of clusters) and NSQN (for 4 clusters) (S6 Fig). The recovery of a signal in these cases is in accordance with the results in the supervised setting. Overall, these results confirm the findings of [31] that network-based normalisation with NSQN allows stratifying patients better than the raw mutation profiles, and also show that the stratification obtained from NetNorM normalisation is both more stable and more clinically relevant than the one obtained with NSQN.

### Patient stratification with randomised networks

**Fig 8:**
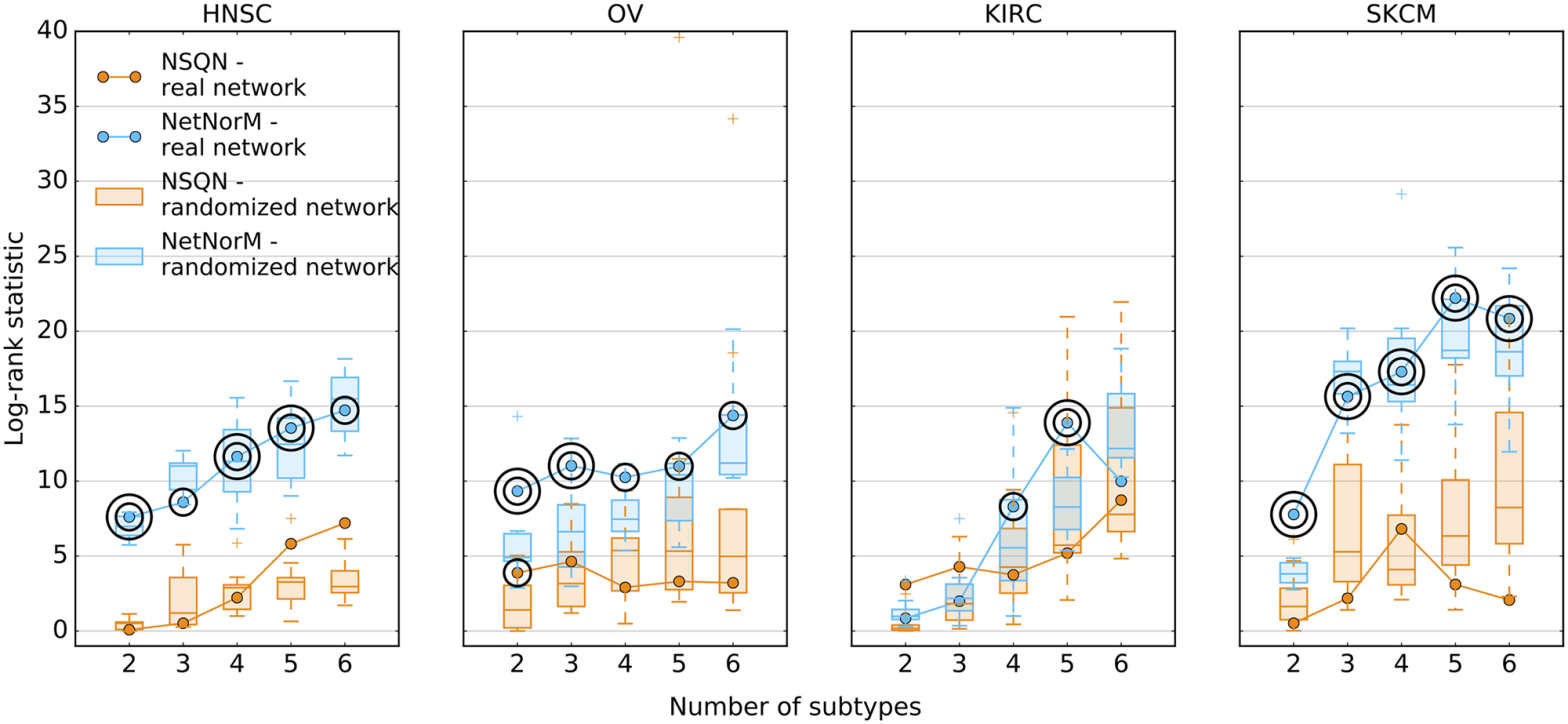
Effect of network randomisation on patient stratification. Log-rank statistic obtained with Pathway Commons (curve) and 10 randomised versions of Pathway Commons (boxplots) with NetNorM (blue) and NSQN (orange) for HNSC, OV, KIRC and SKCM. One circle indicate a P-value *P* ≤ 5 × 10^−2^ and two concentric circles indicate *P* ≤ 1 × 10^−2^.

We now assess whether the biological information contained in Pathway Commons is crucial to obtain subtypes with significantly distinct survival outcomes. For that purpose, we carry out patient stratification with NSQN and NetNorM using 10 randomised versions of Pathway Commons for HNSC, OV, KIRC and SKCM. As for the survival prediction experiment, the randomisation involves shuffling the vertices’ labels so as to keep the structure of the network unchanged. Surprisingly, network randomisation does not affect the log-rank statistic obtained for HNSC and SKCM. This suggests that although NetNorM generates subtypes with more distinct survival times than NSQN for HNSC and SKCM, it does not benefit from Pathway Commons gene-gene interaction knowledge. Rather it exploits the prognostic information contained in the raw mutation profiles as well as the overall mutational burdens as captured by proxy mutations. Regarding KIRC and OV, NetNorM produces subtypes with significantly different survival times with 4 and 5 clusters for KIRC and for any number of clusters for OV. In the case of KIRC, the real network yields the subtypes with the most distinct survival times (N = 5) (Fig 8) while in the case of OV, most randomized networks (at least 15 out of 20 for each number of clusters) produce subtypes with worse association to survival time. This indicates that for KIRC and presumably for OV, NetNorM takes advantage of gene-gene interaction knowledge to stratify patients into clinically relevant subtypes. This is also clearly the case for LUAD with NetNorM when the hyperparameter *k* is set to its value learned by cross-validation in the survival prediction setting (S6 Fig).

### Patient subtypes obtained with NetNorM are characterised by distinct pathways

**Fig 9:**
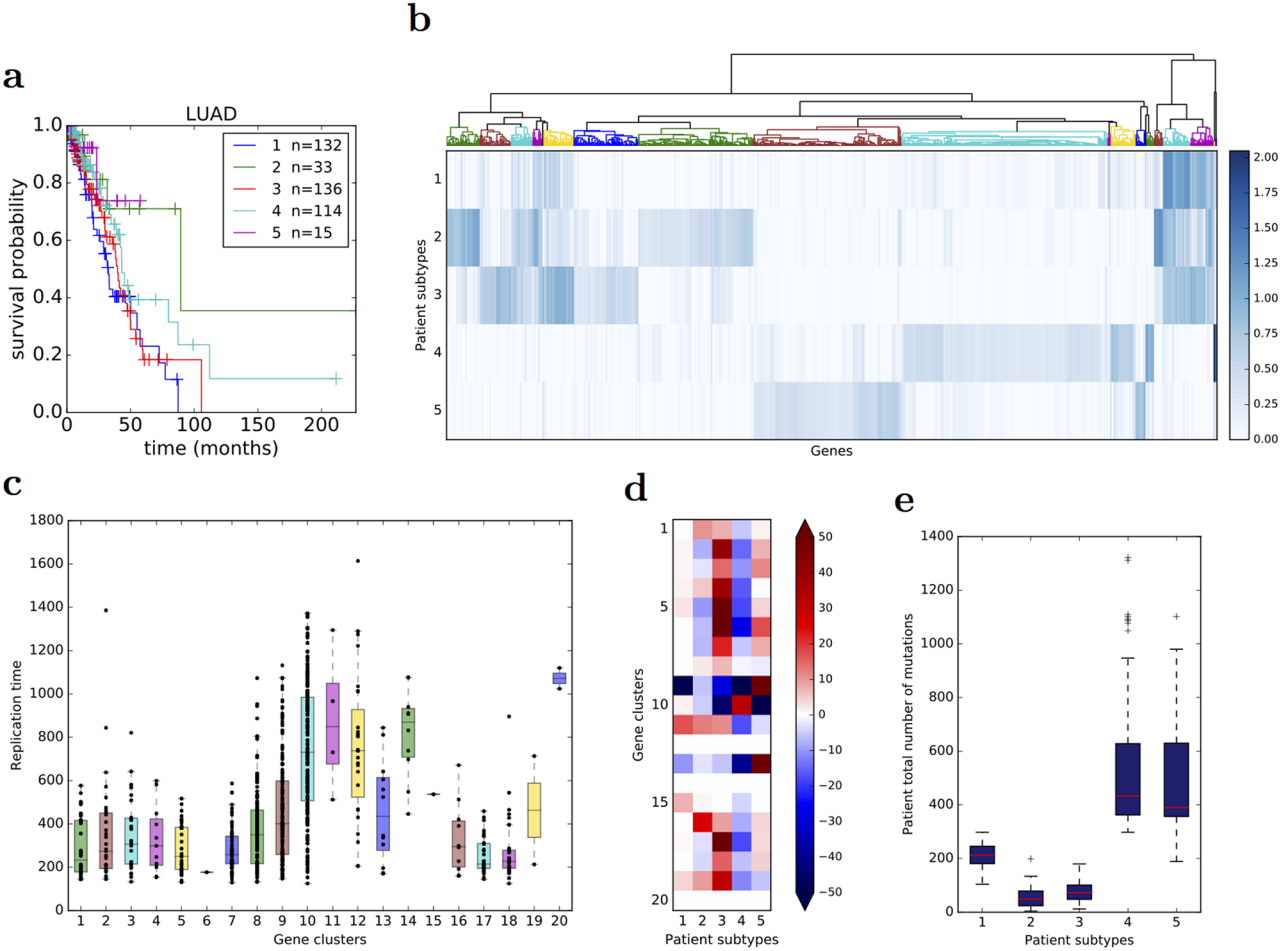
Characterisation of LUAD patient subtypes obtained with NetNorM (*N* = 5 groups, *k* = 315, Pathway Commons). (a) Kaplan Meir survival curves for NetNorM subtypes with significantly distinct survival outcomes. In the legend are indicated the subtype number followed by the number of patients in the subtype. (b) Metapatients matrix obtained by applying NMF to mutation profiles processed with NetNorM. The matrix shown is restricted to the genes with highest variance across metapatients. The genes (columns) are clustered via hierarchical clustering. Clusters are numbered from 1 to 20 from left to right. (c) Distribution of gene replication times across gene clusters. (d) A *χ*^2^ contingency test was performed for each gene cluster to test its enrichment (or depletion) in mutations across patient subtypes given the subtypes’ marginal number of mutations. The value represents the contribution of a subtype to the test statistic, and the colour indicates an enrichment (red) or a depletion (blue) in mutations. (e) Distribution of patients’ total number of (raw) mutations across patient subtypes.

To interpret biologically the subgroups of patients identified by automatic stratification after NetNorM normalisation, we look at differentially mutated genes and pathways across subtypes. We focus on LUAD with N = 5 groups as a proof of principle with *k* set to its value learned by cross-validation in the supervised setting. This choice is motivated by the fact that LUAD is the most promising cancer type for supervised survival prediction and produces interesting results in the unsupervised setting. As the basis vectors or “metapatients” yielded by the NMF summarise the mutational patterns found in the different subtypes, we analyse genes in terms of their weight in the different metapatients, and restrict our attention to the approximately 900 genes displaying highest variance (variance greater than 0.01) across basis vectors since these genes are expected to be the most differentially mutated across subtypes. Interestingly, this gene list comprises most significantly mutated genes in LUAD including *TP53*, *KRAS*, *KEAP1*, *EGFR*, *NF1*, *RB1* [40, 41]. To analyse these genes we cluster them into groups with similar weights across basis vectors using hierarchical clustering (Fig 9b), and we test for enrichment in known biological pathways the 20 gene clusters (GCs) obtained.

One first observation is that the 5 patient subtypes have distinct overall mutational burdens with groups 4 and 5 (resp. 2 and 3) gathering patients with many (resp. few) mutations (Fig 9e). This confirms the fact that NetNorM-normalised profiles contain information about the initial number of mutations, although they are normalised to a fixed number of mutations. More importantly, most GCs exhibit high weights in one metapatient and low weights in others, suggesting that they are mainly enriched in mutations in one single patient subtype (Fig 9b). *χ*^2^ contingency tests (see methods) for each GC confirms that for most of them (17/20), the distribution of the mutations across patient subtypes is not that expected according to subtypes’ overall mutational burdens (*P* < 5 × 10^−2^) (S4 Table). The contribution of each subtype to the test statistic for each GC also confirms that GCs are often enriched in mutations in mainly one patient subtype (Fig 9d). Subtypes could thus easily be associated with one or several GCs, and therefore pathways through pathway enrichment analysis using the KEGG database [54] (see Methods).

Consequently, subtype 3 is characterised by an enrichment in mutations in genes associated with ribosomes and spliceosomes (GCs 2, 3, 4, 5, 6, 7, 8, 17, 18, 19) (S4 Table). Subtype 1 is enriched in mutations in two very small gene clusters (GCs 11 and 16): the first one consists of four genes including *KRAS* and the second one only includes *MUC16*. These two subtypes are those with poorest survival probability. Subtype 4 is mainly enriched in late replicating genes (GC 10) (Fig 9c). This reflects the fact that subtype 4 is enriched in highly mutated patients as there exists a positive correlation between somatic mutation frequency and genes replication time [16]. Subtype 2 is enriched in mutations in genes related to endocytosis and phagosomes (GCs 16, 1, 11). Finally, subtype 5 is very strongly associated with gene clusters 9 and 13. Gene cluster 9 is enriched in genes from the cAMP and PI3K-Akt signaling pathways. Gene cluster 13 could not be significantly associated to a known biological pathway. However it contains *FANCD2* (Fanconi Anemia Complementation Group D2) which is involved in double-strand breaks DNA repair and the maintenance of chromosomal stability [55]. We note that 12 of the 15 patients in subtype 4 present the same 4-nucleotides splice site deletion in *FANCD2*, whereas across the rest of the 430 patients *FANCD2* is mutated in 6 patients only, and only one of these 6 mutations is the same as that observed in subtype 4 patients.

## Discussion

Exploiting the wealth of cancer genomic data collected by large-scale sequencing efforts is a pressing need for clinical applications. Somatic mutations are particularly important since they may reveal the unique history of each tumour at the molecular level, and shed light on the biological processes and potential drug targets dysregulated in each patient. Standard statistical techniques for unsupervised classification or supervised predictive modelling perform poorly when each patient is represented by a raw binary vector indicating which genes have a somatic mutation. This is both because the relevant driver mutations are hidden in the middle of many irrelevant passenger mutations, and because there is usually very little overlap between the somatic mutation profiles of two individuals. NetNorM aims to increase the relevance of mutation data for various tasks such as prognostic modelling and patient stratification by leveraging gene networks as prior knowledge.

**Fig 10:**
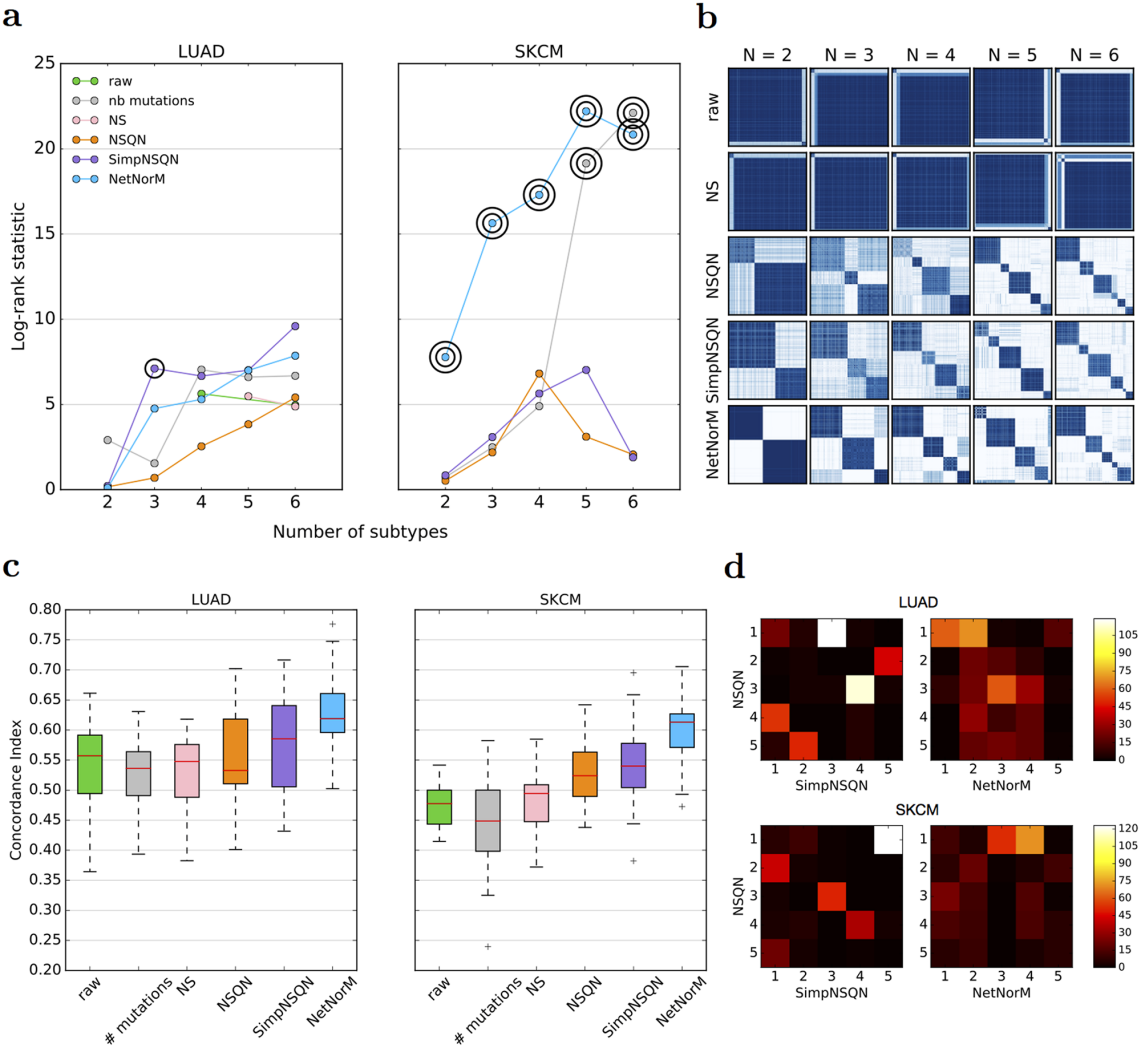
Exploring NSQN and NetNorM performances levers. (a) Subtypes log-rank statistic obtained for LUAD (left) and SKCM (right). One circle indicate a P-value *P* ≤ 5 × 10^−2^ and two concentric circles indicate *P* ≤ 1 × 10^−2^ (log-rank test). (b) Consensus clustering matrices for LUAD. (c) Survival prediction performances for LUAD (left) and SKCM (right). (d) Confusion matrices for LUAD (top) and SKCM (bottom) comparing the subtypes obtained with NSQN and SimpNSQN on the one hand, and NSQN and NetNorM on the other hand. (a, b, c, d) were obtained with Pathway Commons.

One important aspect of NetNorM is the property that, after normalisation, all patients have the same number of 1’s in their normalised mutation profile. Although there is no biological rational for this constraint, we believe that the fact that all normalised samples have the same distribution of values is an important property for many high-dimensional statistical methods such as survival models or clustering techniques to work properly. To support this claim, we notice that the Network-based stratification (NBS) method proposed in [31] performs a quantile normalisation step after network smoothing. To investigate whether the quantile normalisation step in NSQN plays an important role, we applied network smoothing without quantile normalisation (called NS) and performed survival prediction and patients stratification with this representation of the mutations. Surprisingly, NS does not improve over the raw mutation profiles for both LUAD and SKCM (Fig 10c). Moreover just as the raw data, NS is unable to stratify patients into approximately balanced clusters (Fig 10b). This suggests that quantile normalisation plays a crucial role in the performances obtained with NSQN, in spite of non obvious biological justification for this step.

Another important difference between NSQN and NetNorM is the fact that NetNorM only exploits mutation information about direct neighbours in the network, while NSQN can potentially diffuse a mutation further than the direct neighbours. However, we found that NSQN does not benefit from this possibility. Indeed, we tested a simplified version of NSQN where the network propagation is stopped after one iteration, and assessed the performance of the corresponding method which we call SimpNSQN. For survival prediction, we observe no significant difference between NSQN and SimpNSQN (Fig 10c). For patient stratification, SimpNSQN produces subtypes that are vey similar to those produced by NSQN (Fig 10d). Therefore the subtypes generated by both methods associate equally well to clinical data, and even slightly better for SimpNSQN in the case of LUAD (Fig 10a). Overall, these pieces of information indicate that the useful information created by NSQN is mostly concentrated on shared mutated order 1 neighbourhoods, and explain why we observe no loss in performance with NetNorM which explicitly restricts the diffusion of mutations to direct neighbours only. More generally, these elements also indicate that diffusion to indirect neighbours is still difficult with current methods. This is a likely consequence of the small world property of biological graphs [56]. Because the path between any two genes is usually short, diffusion even to order-2 neighbours reaches a substantial number of genes, and therefore the resulting signal observed for one gene is the superposition of a large number of signals originating from close mutations.

NetNorM encodes information about patients’ total number of mutations in the raw data, and potentially can exploit it if this information is relevant for the problem at hand. However we found that the total number of mutations is a poor predictor or survival (Fig 10c), and a poor feature for LUAD patient stratification (Fig 10a). This confirms that NetNorM conserves useful information regarding both the total mutational burden of a patient and the distribution of the mutations on the gene network, and manages to leverage both types of information. In addition to mutational burdens, NetNorM also encodes information about genes’ NMB which proved to carry some prognostic power. The fact that NMB might reveal new insights into mutation profiles is an emerging idea supported by this study. Further support has been formalised with two recently published methods [57, 58] which rely on NMB to achieve state-of-the-art performances for cancer gene discovery.

We emphasize that randomised gene networks lead to significantly worse performances than the real network for survival prediction as well as for patient stratification for several cancers. While it is not always clear whether incorporating gene networks as prior knowledge does help for a given task, this provides a sound argument that such prior knowledge is effectively leveraged with NetNorM.

Increasing the relevance of mutation data to various tasks is a broad project and NetNorM could be extended in many ways. First, although NetNorM was successful for LUAD and SKCM, we note that the method brings few improvements compared to the raw data for the remaining cancer types. Therefore extensive efforts are needed to determine whether it is possible to design representations of mutations that would increase the statistical power of models learned on these datasets. Second, NetNorM does not integrate further information about mutations such as their predicted functional impact. A possible extension could therefore include this type of information. Finally, the distribution of values for the normalised profiles is defined as the mean distribution of the original profiles in the case of NSQN, and simply a binary vector with a fixed number of 1’s in the case of NetNorM, however these choices are empirical. This suggests that an interesting future work may be to assess more precisely the effect of this distribution and, perhaps, optimise it for each specific task.

## Materials and methods

### Patient mutation profiles preprocessing

Whole exome somatic mutation calls (MAF files) were downloaded from TCGA data portal (https://tcga-data.nci.nih.gov/tcga) for 8 cancer types (LUAD, SKCM, GBM, BRCA, KIRC, HNSC, LUSC, OV) (Table 1). The data include point mutations (single nucleotide polymorphism as well as di/tri/oligo-nucleotide polymorphism) and indels. Silent mutations were filtered out and mutations profiles were defined as binary vectors with ones whenever a patient is mutated in a given gene and zeros otherwise.

### Gene-gene interaction network

Pathway Commons (http://www.pathwaycommons.org/pc2/downloads) was used throughout this work (Pathway Commons v6, SIF format). It integrates gene networks from several public databases and aggregates both genetic and protein-protein interactions (PPIs). PPIs refer to physical contacts established between proteins while genetic interactions refer to interactions through regulatory and signalling pathways. To remove interactions involving small molecules in Pathway Commons, the following interaction types were filtered out: “consumption-controlled-by”, “controls-production-of”, “controls-transport-of-chemical”, “chemical-affects”, “reacts-with”, “used-to-produce”, “SmallMoleculeReference”, “ProteinReference;SmallMoleculeReference”, “ProteinReference”. We obtained a network with 16,674 nodes (genes) and 2,117,955 edges (interactions). For the survival prediction task, we also tested the following gene networks: BioGRID v3.4.131, HPRD release 9, HumanNet v1 and STRING v10. For HumanNet and STRING, only the top 10% most confident interactions were retained.

### Network based normalisation of mutation profiles (NetNorM)

NetNorM is a method that integrates patients mutation profiles with a gene network to produce normalised mutation profiles where all patients have the same number *k* of mutations. The target number of mutations *k* is a tuning parameter. In the context of survival prediction (supervised setting), it is learned by cross-validation while for patient stratification (unsupervised setting), it is set as the median number of mutations in a cohort, or alternatively to the median best *k* learned across cross-validation folds for survival prediction. Concretely, NetNorM defines a ranking over genes separately for each patient and then use this ranking to normalise mutation profiles. The ranking defined in NetNorM is obtained with a simple two-step procedure. First, genes are ranked according to their mutation status with mutated genes ranked higher than non mutated genes. Then, mutated genes are ranked according to their degree (i.e. their number of neighbours) and non mutated genes are ranked according to their number of mutated neighbours. The normalisation is then obtained by considering the *k* highest ranked genes as mutated while the rest of the genes will be considered non mutated. By construction, mutated genes are always ranked higher than non-mutated genes. Therefore patients with a lot of mutations will have mutations removed while patients with few mutations will hold artificial proxy mutations. Note that when the obtained ranking contains ties, all genes are given distinct ranks according to the order in which they occur in the mutation matrix.

### Network smoothing with quantile normalisation (NSQN)

Network smoothing propagates the influence of mutations over gene-gene interaction networks. It was implemented according to the following update function [31]:

$$\begin{array}{c} X_{t+1}=\alpha X_{t}D^{-\frac{1}{2}}\;AD^{-\frac{1}{2}}+\left(1-\alpha \right)X_{0} \end{array}$$

where ***X*_*t*_** is the patient × genes mutation matrix at iteration *t*, ***X*_0_** is the initial binary mutation matrix, ***A*** is the adjacency matrix representing the network used and ***D*** is the diagonal degree matrix where $D_{ii}=\sum_{j}A_{ij}$. *α* is a tuning parameter controlling the length of diffusion paths over the network. Similarly to the parameter *k* in the context of NetNorM, it is learned by cross-validation for survival prediction (supervised task) while for patient stratification (unsupervised task) it is set as *α* = 0.5 as recommended in [31] with Pathway Commons or alternatively to the median best *α* learned across survival prediction cross-validation folds. The update function is applied until convergence, and the resulting smoothed matrix is then quantile normalised so that all patients have the same mutation distribution.

### Simplified version of NSQN (SimpNSQN)

The simplified version of NSQN does not propagate mutations further than to order 1 neighbours in the network. More precisely, the SimpNSQN score of a gene is equal to its number of mutated neighbours normalised by its degree and by the degrees of its neighbours, plus a constant if the gene is mutated. This is obtained by computing:

$$\begin{array}{c} X=\alpha X_{0}D^{-\frac{1}{2}}\;AD^{-\frac{1}{2}}+\left(1-\alpha \right)X_{0} \end{array}$$

where ***X*_0_** is the initial binary mutation matrix, ***A*** is the adjacency matrix representing the network used, ***D*** is the diagonal degree matrix where $D_{ii}=\sum_{j}A_{ij}$ and α∈R is a tuning parameter. Note that SimpNSQN uses the same update equation as NSQN but it is run only once.

### Sparse survival SVM

To estimate a survival model from high-dimensional mutation profiles, we use a survival SVM model [59] combined with a sparsity-inducing regularisation to automatically perform gene selection. Let *δ*_*i*_ = 1 (resp. *δ*_*i*_ = 0) if patient *i* is deceased (resp. censored), and $y_{i}\in R$ be the observed survival time of patient *i*. It corresponds to either a failure or a censoring time depending on whether the patient is deceased or censored. Define *Z* ∈ {0, 1}^*n*×*n*^ which indicates whether a pair of patients is comparable, i.e,

$$\begin{array}{c} Z_{ij}=\{\begin{array}{c} \text{1}\;\text{if}\;\left(y_{i}<y_{j}\;\text{and}\;\delta_{i}=1\right)\;\text{or}\;\left(y_{j}<y_{i}\;\text{and}\;\delta_{j}=1\right)\;,\\ \text{1}\;\text{if}\;(y_{i}=y_{j}\;\text{and}\;\left(\delta_{i}=1\;\text{or}\;\delta_{j}=1\right))\;,\\ \text{0}\;\text{otherwise}\;\text{.} \end{array} \end{array}$$

Finally, let ***x*_*i*_** ∈ {0, 1}^*p*^ be the mutation profile of patient *i*. The survival time of patient *i* is modelled as *s*_*i*_ = ***w*^*T*^*x*_*i*_** where $w\in R^{p}$ is the model parameter learned using ranking Support Vector Machines (rSVM) as in [59]. However to get a sparse ***w*** we introduce an *ℓ*_1_ regularisation instead of the *ℓ*_2_ regularisation in [59] and thus solve the following optimisation problem:

$$\begin{array}{c} \underset{w}{\text{minimise}\;}\frac{1}{2}{\left||w|\right|}_{1}+C\sum_{i,j}Z_{ij}\;\ell_{hinge}\left(w^{T}\left(\;x_{j}-x_{i}\right)\right)\;, \end{array}$$

where *ℓ*_*hinge*_(*u*) = max(1 − *u*, 0) is the hinge loss and C∈R is the regularisation parameter. To solve this problem we used the support vector classification algorithm *svm.LinearSVC* from the Python package *scikit learn* [60]. This optimisation problem maximises a convex relaxation of the Concordance Index (CI) which measures how well the predicted survival times ***s*** are in accordance with the observed survival times ***y*** for the comparable pairs of patients. Formally, $\text{CI}=\frac{1}{\left|Z\right|}\sum_{y_{i}\leq y_{j}}Z_{ij}I\left(s_{j}-s_{i}\right)$ where

$$\begin{array}{c} I\left(x\right)=\{\begin{array}{c} \text{1}\;\text{if}\;x>0\;,\\ \frac{1}{2}\;\text{if}\;\text{x}=\text{0}\;,\\ \text{0}\;\text{otherwise}\;\text{,} \end{array} \end{array}$$

and $\left|Z\right|=\sum_{y_{i}\leq y_{j}}\;Z_{ij}$. To evaluate the CI obtained on a given dataset, samples were split in 80% train and 20% test sets 20 times using 4 five-fold cross-validation. Each time, a model was learned on the training set and tested on the test set. The CI was computed according to a python implementation of the function *estC* from the R package *compareC*. Hyperparameters were learned thanks to an inner 5-fold cross-validation on the training set. The values tested for *C* ranged from 1 × 10^−4^ to 1 × 10^2^ included in log scale. The values tested for *α* ranged from 0.1 to 0.9 included with steps of 0.1. Finally the values tested for *k* were chosen to span a grid from *k*_*min*_ and *k*_*max*_ with steps of 2, where *k*_*min*_ and *k*_*max*_ are the first and third quartiles of the distribution of patients’ total number of mutations. *k*_*min*_ and *k*_*max*_ differ for each cohort (S2 Table).

### Patient stratification

Let $X\in R^{n\times p}$ be the matrix with patient mutations profiles as rows. To cluster the patients we perform a non-negative matrix factorisation (NMF) on ***X***, i.e., solve the following optimisation problem:

$$\begin{array}{c} \underset{W,H>0}{\text{minimise}\;}||X-{WH||}_{2}^{2}\;, \end{array}$$

where $H\in R^{N\times p}$ defines *N* basis vectors or “metapatients” and $W\in R^{n\times N}$ defines basis vectors loadings. Patient *i* was then assigned to the group *j* ∈ {1‥*N*} that represents him best i.e. $\underset{j}{argmax}\;W_{ij}$. To promote robust cluster assignments, NMF was applied 1000 times to subsamples of the dataset composed of 80% of the samples and 80% of the features chosen at random without replacement. A consensus matrix $C\in R^{n\times n}$ was then derived from the 1000 cluster assignments obtained where each entry *C*_*ij*_ corresponds to the frequency at which two patients where clustered in the same group over all samplings where both patients were retained. The final cluster assignment was obtained by applying hierarchical clustering to the consensus matrix with euclidean distance and average linkage.

To assess the stability of the obtained clusters, we computed the proportion of ambiguous clustering (PAC) which is the proportion of discordant cluster assignments obtained through consensus clustering. Cluster assignments for a pair of patients (*i*, *j*) were considered discordant when 0.25 ≤ *C*_*ij*_ ≤ 0.75.

In the case where only the total number of mutations was used for stratification, NMF is not applicable and kMeans was used instead with 1000 restarts and initialisation by kMeans++ [61].

### Analysis of the proxy genes selected by the sparse survival SVM with NetNorM

Several proxy genes have a prognostic power according to log-rank tests performed for each gene separately and which compare patients with mutations (proxy or not) versus those without (*P* ≤ 1 × 10^−2^). The difference in survival outcomes observed may be due to at least two types of information encoded in proxy genes: patients’ overall mutational burden and genes’ neighbourhood mutational burden (NMB). To clarify the contributions of each effect, we investigate whether such distinct survival outcomes can be obtained with proxies for the total number of mutations only, regardless of NMBs. To this end, we simulate proxy mutations for each gene separately according to a model that only depends on patients’ total number of mutations. Let $T_{i}\in N$ be the total number of mutations of patient *i*, *i* ∈ {1, …, *n*}. Let $M_{o}\subset \left\{1,...,n\right\}$ and $M_{p}\subset \left\{1,...,n\right\}$ indicate which patients have original and proxy mutations respectively. For a given proxy gene whose mutations are described by the sets *M*_*o*_ and *M*_*p*_, we leave the original mutations untouched and reallocate the proxy mutations according to

$$\begin{array}{c} P\left(i\in M_{p}|T_{i}\right)=\{\begin{array}{c} 0\;\text{if}\;\left(T_{i}\geq k\right)\;\text{or}\;\left(i\in M_{o}\right)\\ \frac{k-T_{i}}{\alpha }\;\text{otherwise} \end{array} \end{array}$$

where *α* is chosen so that the probabilities sum to 1. Proxy mutations are drawn from this model 1000 times. Each time we compute the log-rank statistic between the mutated and non mutated patients which yields a distribution of the log-rank statistic under the null hypothesis. The actual log-rank statistic obtained using NetNorM is then compared to this distribution to accept or reject the null hypothesis. Rejecting the null hypothesis means that the difference in survival outcomes observed between the patients with and without artificial mutations is not only driven by patients’ total number of mutations.

### Survival analysis using patient subtypes and clinical data

To determine whether the obtained patient subtypes are predictive of survival beyond clinical data, we fitted a Cox proportional hazards regression model to the clinical data and to the clinical data augmented with a variable describing patients’ subtypes. We then performed a likelihood ratio test to compare the two models. The clinical variables used were downloaded from TCGA. It includes age, gender, stage, extent of spread to the lymph nodes, presence of metastasis, histology for both LUAD and SKCM and further variables such as smoking history, history of prior malignancy, residual tumour after surgery, tumour dimensions for LUAD and clark level at diagnosis, primary melanoma mitotic rate, new tumour event after initial treatment (yes/no), primary melanoma tumour ulceration (yes/no), primary melanoma known (yes/no) for SKCM.

### Identifying differentially mutated genes and pathways across subtypes

We obtain gene clusters by applying hierarchical clustering with centroid linkage and Euclidean distance to the columns of the metapatients matrix (restricted to high variance genes). To obtain a reasonable number of gene clusters to analyse, we cut the hierarchical cluster tree at a distance threshold of 5.5, yielding 20 clusters. Gene clusters can be categorised into two types: those that contain a lot of proxy mutations (≥ 80% of the total mutational load of the cluster) and whose genes form a dense subgraph, and those that have neither of these two features. The presence of dense subgraphs with many proxy mutations results from the fact that NetNorM tends to add proxy mutations to all genes in a dense subgraph or none since they all have roughly the same number of mutated neighbours. The association of a gene cluster with one subtype can therefore indicate two things: either the subtype is expected to be enriched in proxy mutations in the corresponding gene cluster, which in turn indicates that the subgraph in which the cluster lies is expected to be enriched in mutations, or the gene cluster itself is expected to be enriched in mutations in the corresponding subtype. The enrichment or depletion in mutations of one gene cluster across patient subtypes was therefore tested slightly differently according to the gene cluster type. In the first case, we first define the neighbourhood of the gene clusters as all genes lying in the same dense subgraph. Specifically, we include in the subgraph all genes sharing an edge with at least 90% of the genes in the cluster, thus keeping subgraphs very dense. The obtained set of genes is the one tested for enrichment in mutations across subtype. In the second case, the gene cluster is directly tested for enrichment. Enrichment is assessed with a *χ*^2^ contingency test, where the contingency table is defined by the following marginals: the total number of raw mutations in each subtype, and the total number of raw mutations in and outside the gene cluster (generalised to the embedding of a dense subgraph if it is relevant).

Gene clusters are searched for pathway enrichment using DAVID online tool [62] (https://david.ncifcrf.gov/summary.jsp) with the KEGG database [54]. They are also tested for enrichment in late replicating genes thanks to a permutation test using data downloaded from http://www.broadinstitute.org/cancer/cga/mutsig_run. For each gene cluster *c* of length *l*_*c*_, *l*_*c*_ genes are chosen uniformly at random without replacement from the list of genes with replication time information. This sampling is performed 1000 times and the null distribution was obtained by computing the median replication time of these 1000 gene sets. The median replication time of cluster *c* is then compared to the null distribution to yield a p-value, i.e. the probability to observe a set of genes of length *l*_*c*_ with median replication time at least as extreme.

## Supporting information

S1 Fig Effect of silent mutations on the survival predictive power of the raw mutation profiles, and mutation profiles processed with NSQN and NetNorM (with Pathway Commons as gene network). In the legend, ‘Filtered silent’ indicates that genes with silent mutations were not considered as mutated while ‘with silent’ indicates that genes with silent mutations were considered as mutated. For each cancer type, samples were split 20 times in training and test sets (4 times 5-fold cross-validation). Each time a sparse survival SVM was trained on the training set and the test set was used for performance evaluation. Wilcoxon signed rank tests were run to compare the performances obtained with and without silent mutations for each method and cancer type. Resulting *P*-values below 0.05 or 0.01 are indicated with asterisks (*P* < 5 × 10^−2^ (*) or *P* < 1 × 10^−2^ (**)).
(TIFF)

S2 Fig Survival predictive power of the mutation profiles processed with NSQN and NetNorM assessed with five different gene-gene interaction networks: Pathway Commons, BioGRID, HPRD, STRING and HumanNet. For STRING and HumanNet, only the top 10% most confident interactions were kept in the network. The performances obtained with the raw data slightly vary according to the network used since only the genes present in the network are considered. For each cancer type, samples were split 20 times in training and test sets (4 times 5-fold cross-validation). Each time a sparse survival SVM was trained on the training set and the test set was used for performance evaluation. The presence of asterisks indicate when the test CI is significantly different between 2 conditions (Wilcoxon signed rank test, *P* < 5 × 10^−2^ (*) or *P* < 1 × 10^−2^ (**)).
(TIFF)

S3 Fig Comparison of the survival predictive power of: the most predictive gene, the raw mutation data, NSQN and NetNorM (with Pathway Commons as gene network) for 8 cancer types. For each cancer type, samples were split 20 times in training and test sets (4 times 5-fold cross-validation). In the case where only one gene was used to predict survival, the gene with the best concordance index on the training set was chosen and its performance evaluated on the test set. Otherwise, each time a sparse survival SVM was trained on the training set and the test set was used for performance evaluation. The presence of asterisks indicate when the test CI is significantly different between 2 conditions (Wilcoxon signed rank test, *P* < 5 × 10^−2^ (*) or *P* < 1 × 10^−2^ (**)).
(PDF)

S4 Fig Survival predictive power of mutation data preprocessed according to five different schemes: 1) the raw data concatenated with a feature (scaled to unit variance) recording the total number of mutations in each patient (light gray); 2) the raw data concatenated with a feature called ‘proxies’ (scaled to unit variance) which is equal to 0 if the patient has more than *k* mutations (*k* is learned by cross-validation) and is equal to the total number of mutations otherwise (light purple), 3) the NetNorM representation concatenated with ‘proxies’ (purple) scaled to unit variance; 4) the raw binary mutation profiles; 5) mutation profiles processed with NSQN (orange); 6) mutation profiles processed with NetNorM (blue). Pathway Commons was used with NetNorM and NSQN. Samples were split 20 times in training and test sets (4 times 5-fold cross-validation). Each time a sparse survival SVM was trained on the training set and the test set was used for performance evaluation.
(PDF)

S5 Fig Survival predictive power of mutation data (raw binary mutations, mutations preprocessed with NSQN or NetNorM with Pathway Commons), clinical data, and the combination of both for LUAD and SKCM. The combination of both data types was obtained by concatenating the mutation features with the clinical features scaled to unit variance. For both cancers, samples were split 20 times in training and test sets (4 times 5-fold cross-validation). Each time a sparse survival SVM was trained on the training set and the test set was used for performance evaluation.
(TIFF)

S6 Fig Patient stratification based on NetNorM (resp. NSQN) with hyperparameter *k* (resp. *α*) set to the value learned cross-validation for the survival prediction task instead of the default value. The stratification was obtained using NMF with consensus clustering. (a) Effect of network randomisation on patient stratification. Log-rank statistic obtained with Pathway Commons (curve) and 10 randomised versions of Pathway Commons (boxplots) with NetNorM (blue) and NSQN (orange) for LUAD and SKCM. One circle indicate a P-value *P* ≤ 5 × 10^−2^ and two concentric circles indicate *P* ≤ 1 × 10^−2^. (b) Kaplan Meir survival curves for NetNorM subtypes with significantly distinct survival outcomes (we illustrated the case with 5 subgroups for both LUAD and SKCM). In the legend are indicated the subtype number followed by the number of patients in the subtype.
(TIFF)

S1 Table Summary of the genes selected when only one gene is used to predict survival. For each gene the number of folds (out of 20 folds) where the gene is selected is indicated.
(TIFF)

S2 Table Statistics of the distributions of patients’ total number of mutations for each cancer. Only mutations in genes present in Pathway Commons are taken into account. *Q*1 and *Q*3 refer to the 1^*st*^ and 3^*rd*^ quartiles respectively. The parameter *k* (NetNorM) was learned by cross-validation in the supervised setting using cancer specific cross-validation grids delimited by *Q*1 and *Q*3, and with a step-size of 2.
(TIFF)

S3 Table Summary of the values of *k* (NetNorM) and *α* (NS and NSQN) learned by cross-validation for survival prediction. The values given are the medians obtained over 20 cross-validation folds performed for each dataset and each method.
(TIFF)

S4 Table The gene clusters characterising LUAD patient subtypes obtained with NetNorM (*N* = 5 groups, Pathway Commons). *nb. of genes*: number of genes in a cluster, *subgraph density*: density of the subgraph whose vertices are the genes inside a cluster, *proxy mutations fraction*: number of proxy mutations out the the total number of mutations for a gene cluster across all patients.
(TIFF)
