# Supplementary figures and images for: NetNorM: Capturing cancer-relevant information in somatic exome mutation data with gene networks for cancer stratification and prognosis
Source: PLoS Comput Biol. 2017 Jun 26;13(6):e1005573. doi: 10.1371/journal.pcbi.1005573 (PMC5507468; doi:10.1371/journal.pcbi.1005573)

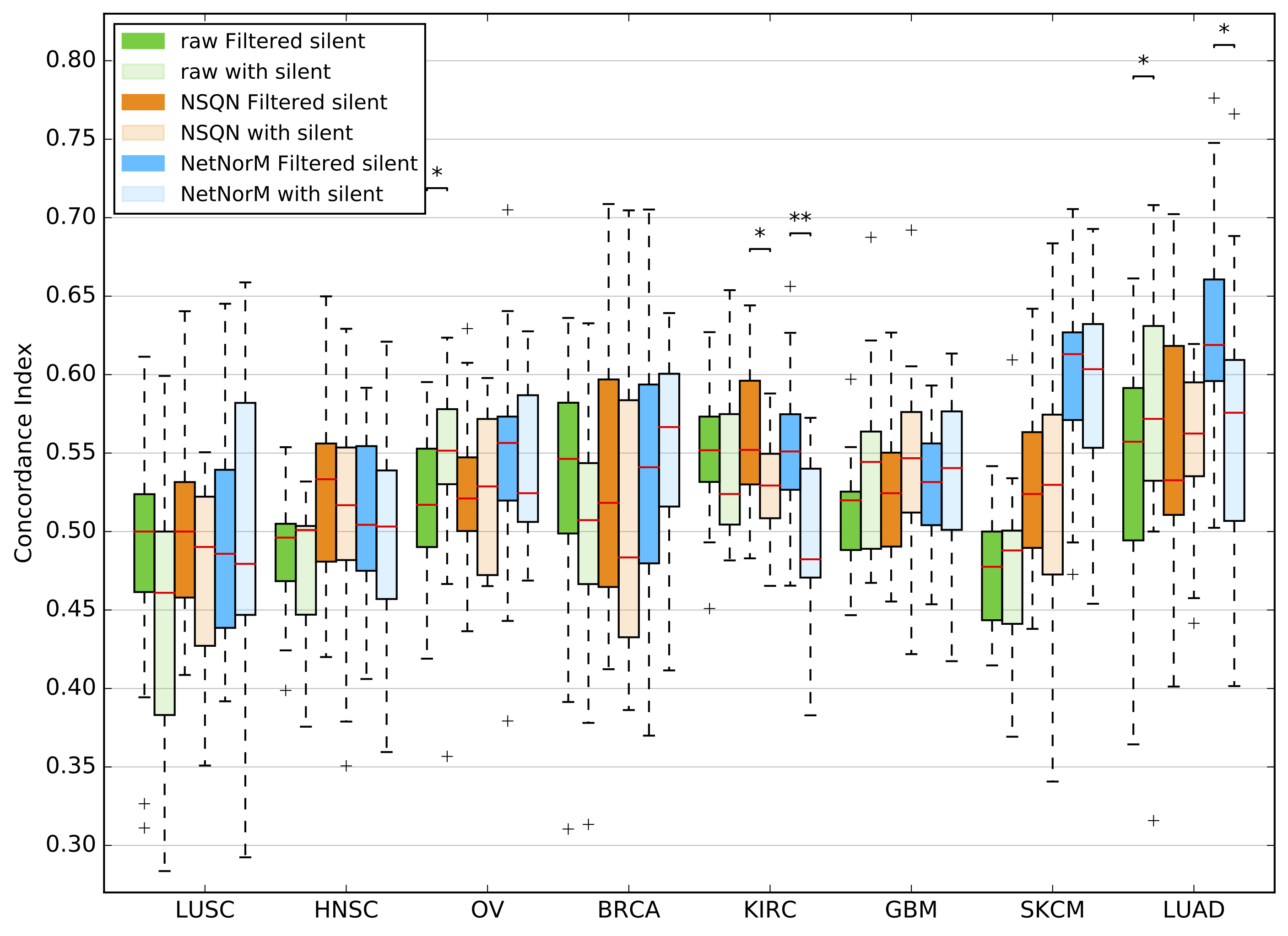

Supplement: S1 Fig — In the legend, ‘Filtered silent’ indicates that genes with silent mutations were not considered as mutated while ‘with silent’ indicates that genes with silent mutations were considered as mutated. For each cancer type, samples were split 20 times in training and test sets (4 times 5-fold cross-validation). Each time a sparse survival SVM was trained on the training set and the test set was used for performance evaluation. Wilcoxon signed rank tests were run to compare the performances obtained with and without silent mutations for each method and cancer type. Resulting P-values below 0.05 or 0.01 are indicated with asterisks (P < 5 × 10^−2^ (*) or P < 1 × 10^−2^ (**)). (TIFF) [file pcbi.1005573.s001.tiff]

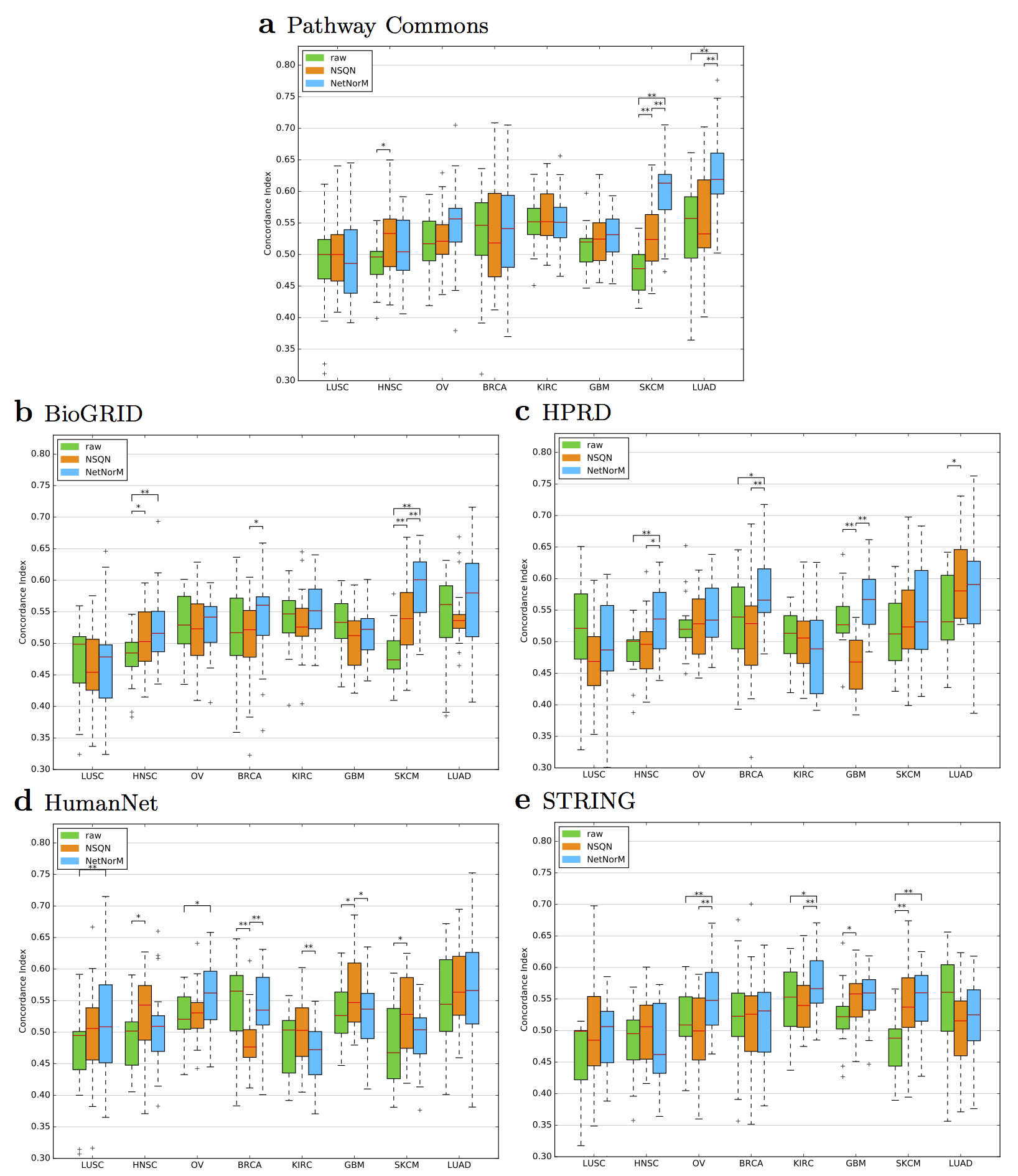

Supplement: S2 Fig — For STRING and HumanNet, only the top 10% most confident interactions were kept in the network. The performances obtained with the raw data slightly vary according to the network used since only the genes present in the network are considered. For each cancer type, samples were split 20 times in training and test sets (4 times 5-fold cross-validation). Each time a sparse survival SVM was trained on the training set and the test set was used for performance evaluation. The presence of asterisks indicate when the test CI is significantly different between 2 conditions (Wilcoxon signed rank test, P < 5 × 10^−2^ (*) or P < 1 × 10^−2^ (**)). (TIFF) [file pcbi.1005573.s002.tiff]

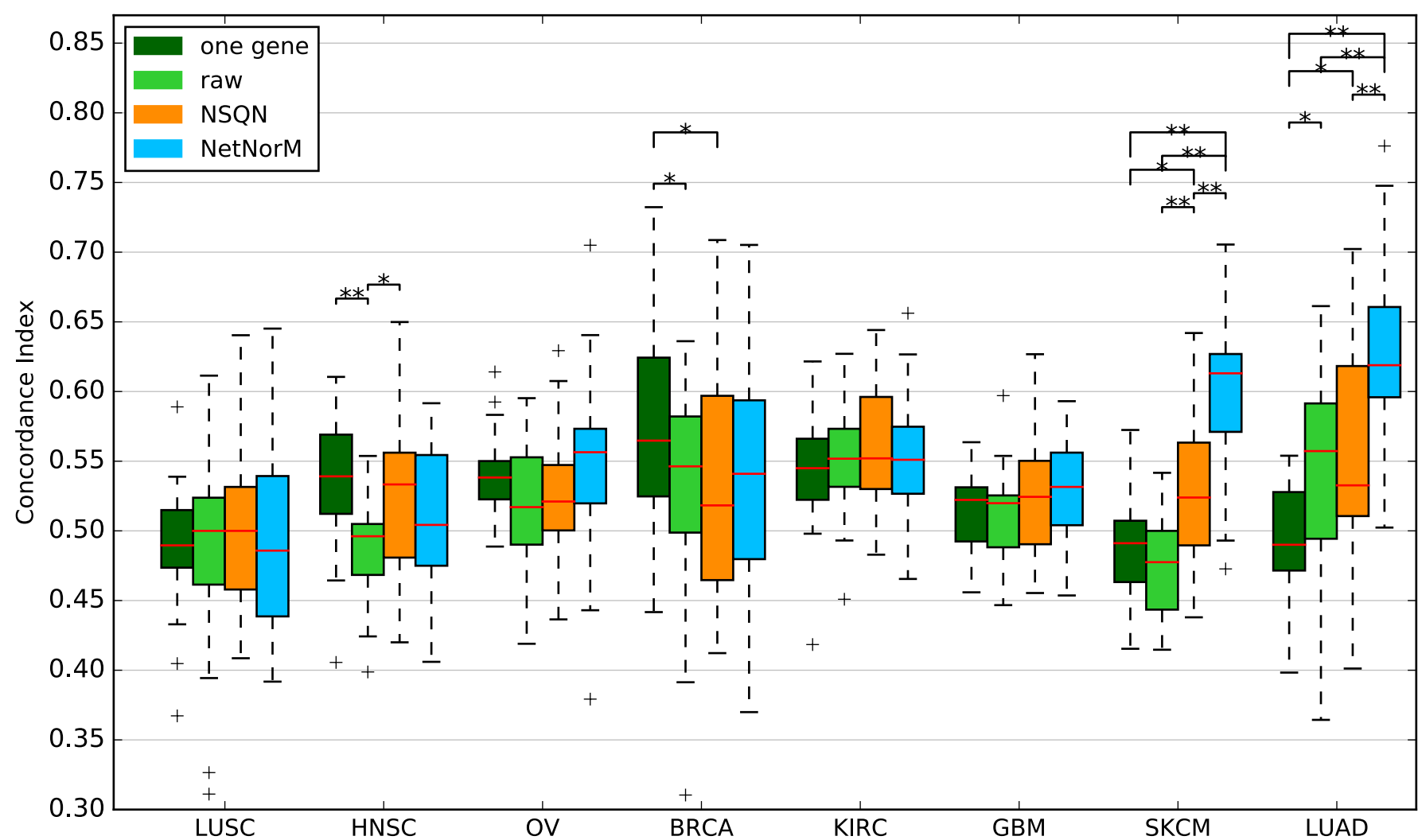

Supplement: S3 Fig — For each cancer type, samples were split 20 times in training and test sets (4 times 5-fold cross-validation). In the case where only one gene was used to predict survival, the gene with the best concordance index on the training set was chosen and its performance evaluated on the test set. Otherwise, each time a sparse survival SVM was trained on the training set and the test set was used for performance evaluation. The presence of asterisks indicate when the test CI is significantly different between 2 conditions (Wilcoxon signed rank test, P < 5 × 10^−2^ (*) or P < 1 × 10^−2^ (**)). (PDF) [file pcbi.1005573.s003.pdf]

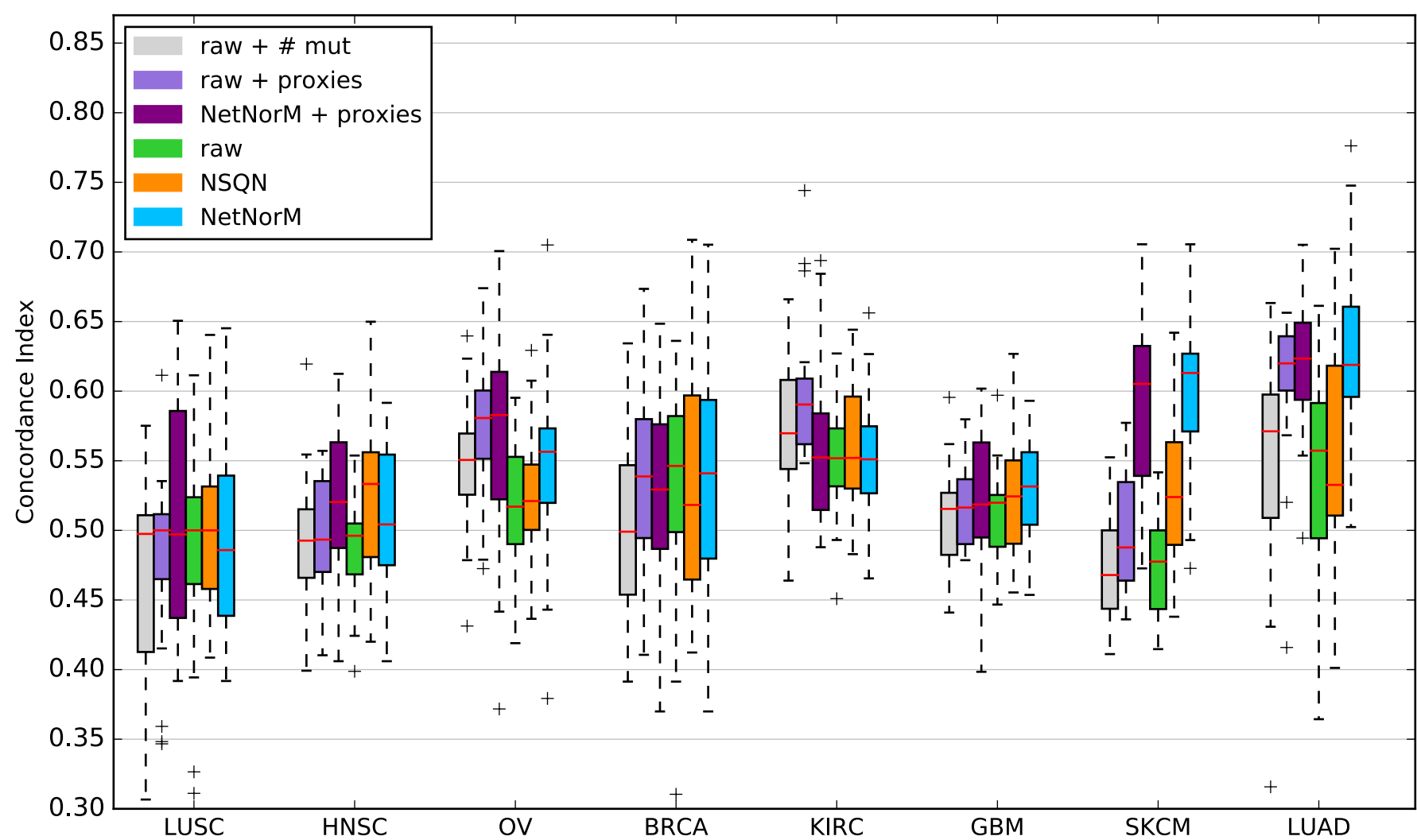

Supplement: S4 Fig — Pathway Commons was used with NetNorM and NSQN. Samples were split 20 times in training and test sets (4 times 5-fold cross-validation). Each time a sparse survival SVM was trained on the training set and the test set was used for performance evaluation. (PDF) [file pcbi.1005573.s004.pdf]

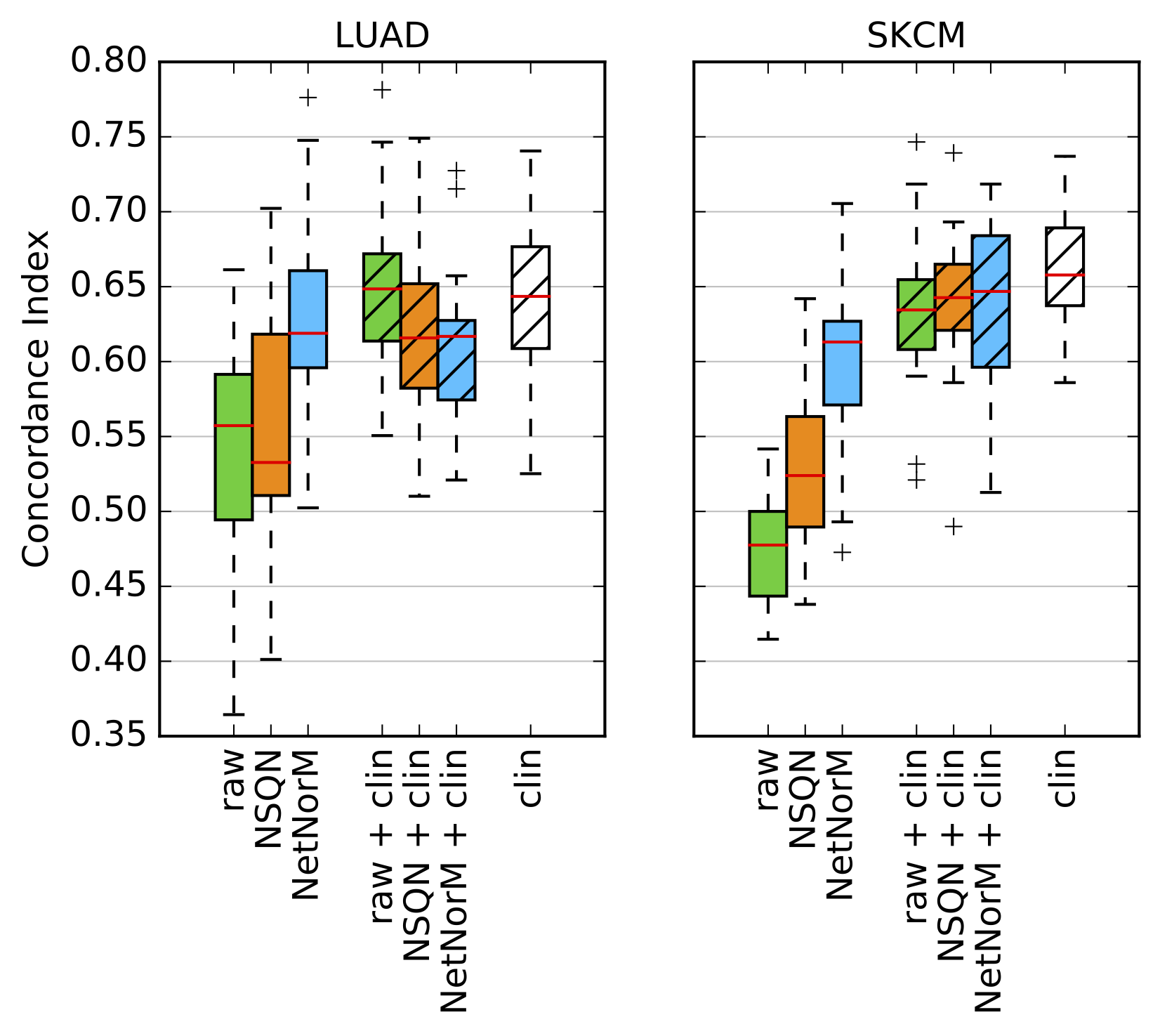

Supplement: S5 Fig — The combination of both data types was obtained by concatenating the mutation features with the clinical features scaled to unit variance. For both cancers, samples were split 20 times in training and test sets (4 times 5-fold cross-validation). Each time a sparse survival SVM was trained on the training set and the test set was used for performance evaluation. (TIFF) [file pcbi.1005573.s005.tiff]

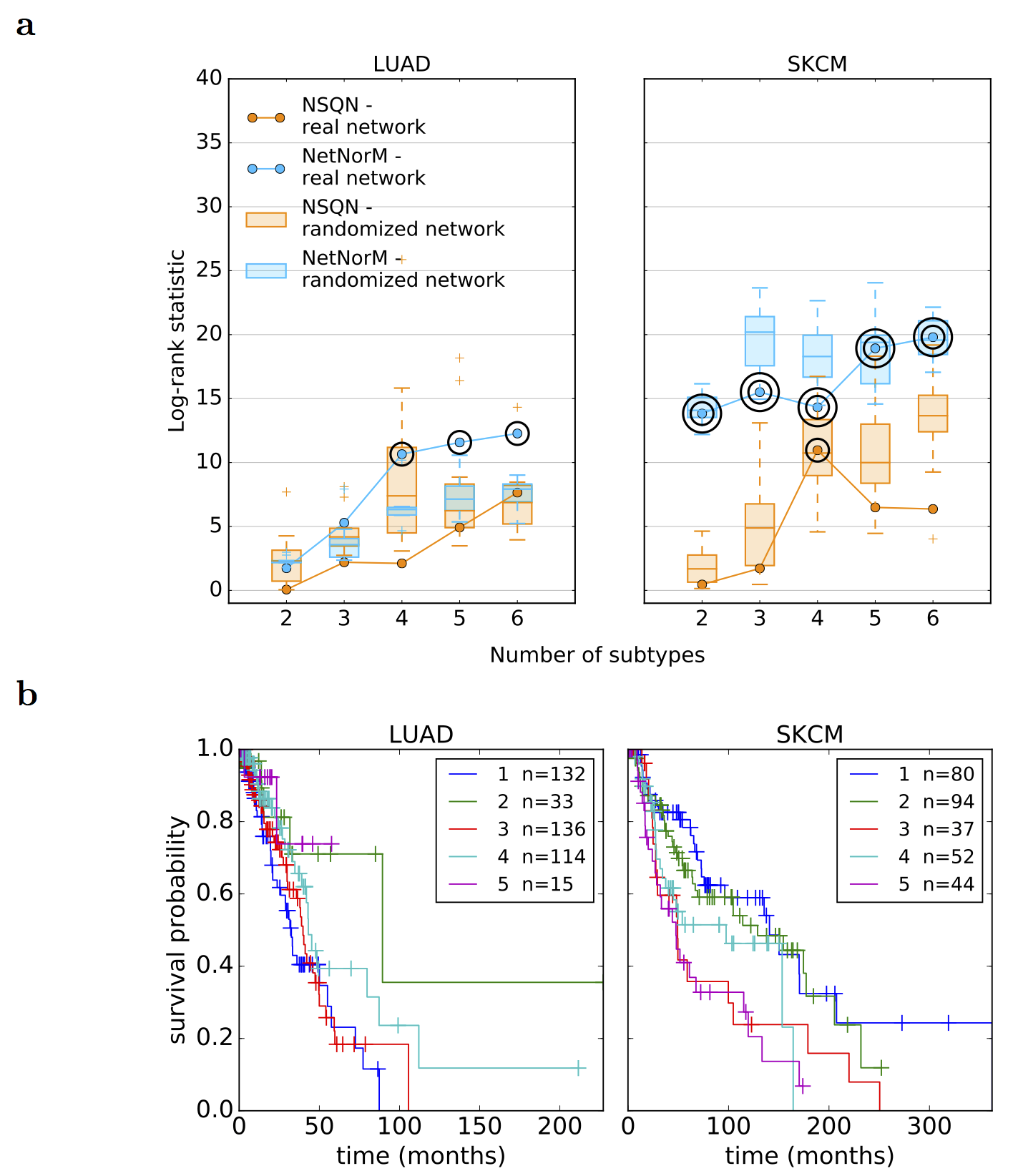

Supplement: S6 Fig — The stratification was obtained using NMF with consensus clustering. (a) Effect of network randomisation on patient stratification. Log-rank statistic obtained with Pathway Commons (curve) and 10 randomised versions of Pathway Commons (boxplots) with NetNorM (blue) and NSQN (orange) for LUAD and SKCM. One circle indicate a P-value P ≤ 5 × 10^−2^ and two concentric circles indicate P ≤ 1 × 10^−2^. (b) Kaplan Meir survival curves for NetNorM subtypes with significantly distinct survival outcomes (we illustrated the case with 5 subgroups for both LUAD and SKCM). In the legend are indicated the subtype number followed by the number of patients in the subtype. (TIFF) [file pcbi.1005573.s006.tiff]

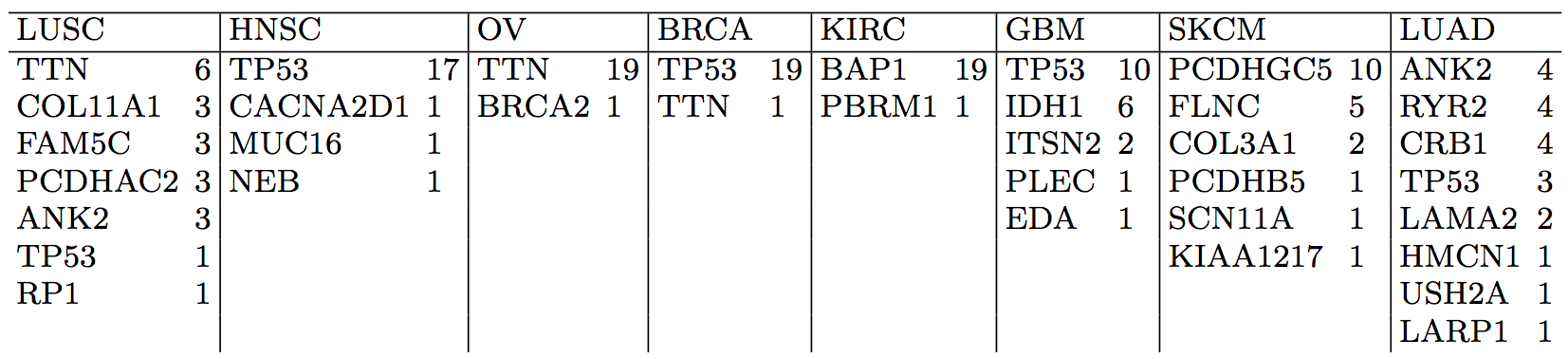

Supplement: S1 Table — For each gene the number of folds (out of 20 folds) where the gene is selected is indicated. (TIFF) [file pcbi.1005573.s007.tiff]

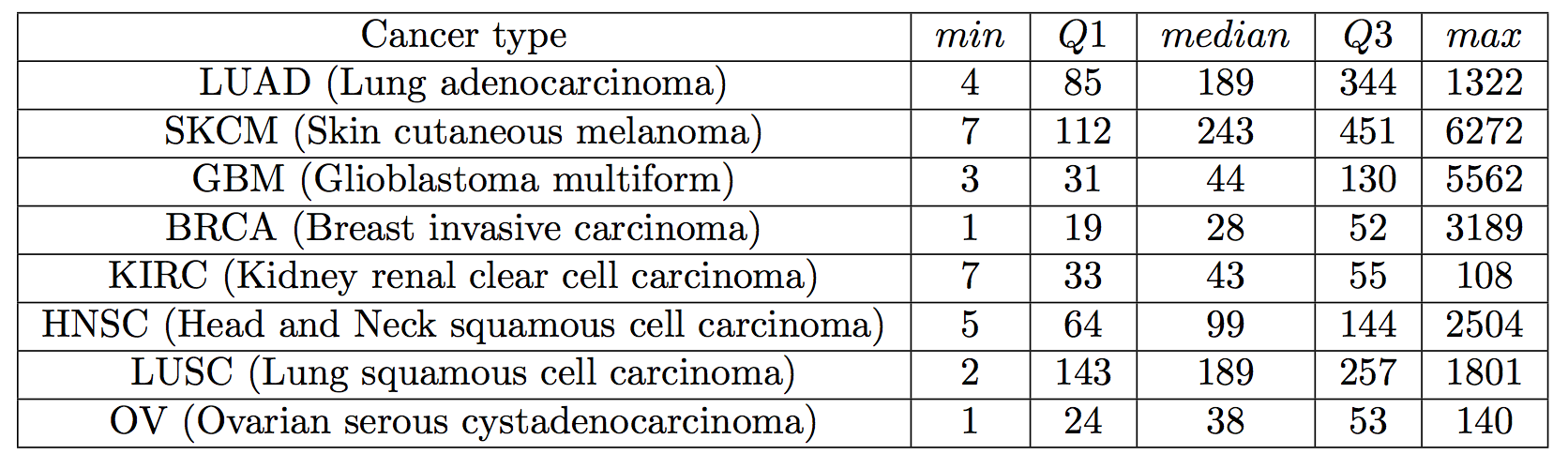

Supplement: S2 Table — Only mutations in genes present in Pathway Commons are taken into account. Q1 and Q3 refer to the 1st and 3rd quartiles respectively. The parameter k (NetNorM) was learned by cross-validation in the supervised setting using cancer specific cross-validation grids delimited by Q1 and Q3, and with a step-size of 2. (TIFF) [file pcbi.1005573.s008.tiff]

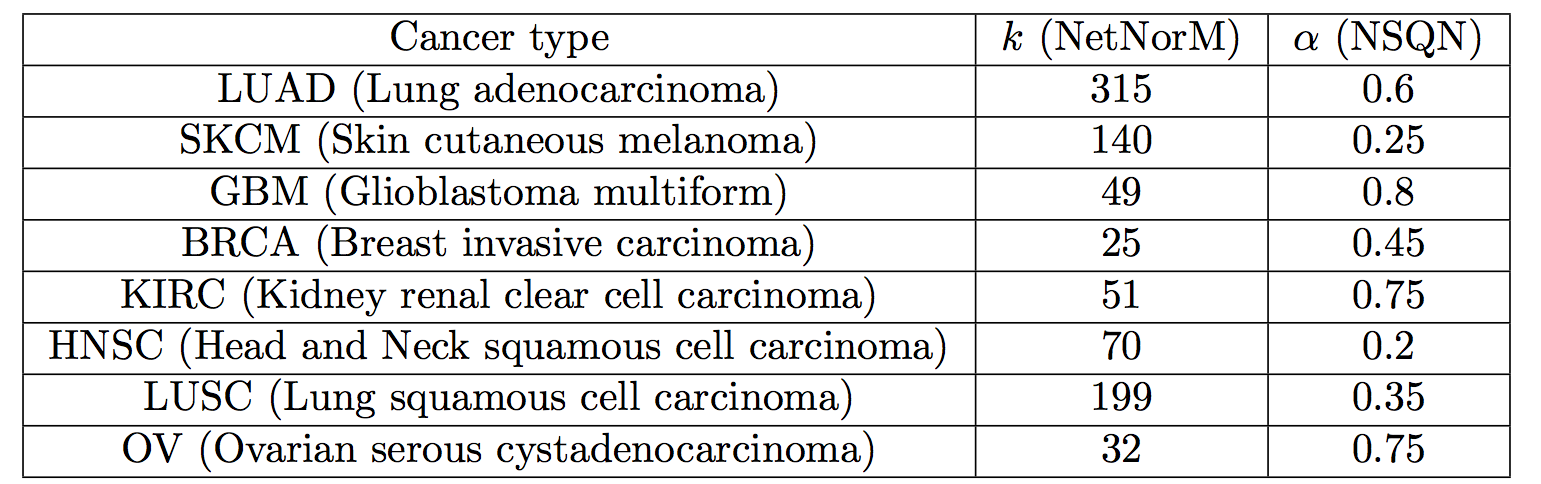

Supplement: S3 Table — The values given are the medians obtained over 20 cross-validation folds performed for each dataset and each method. (TIFF) [file pcbi.1005573.s009.tiff]

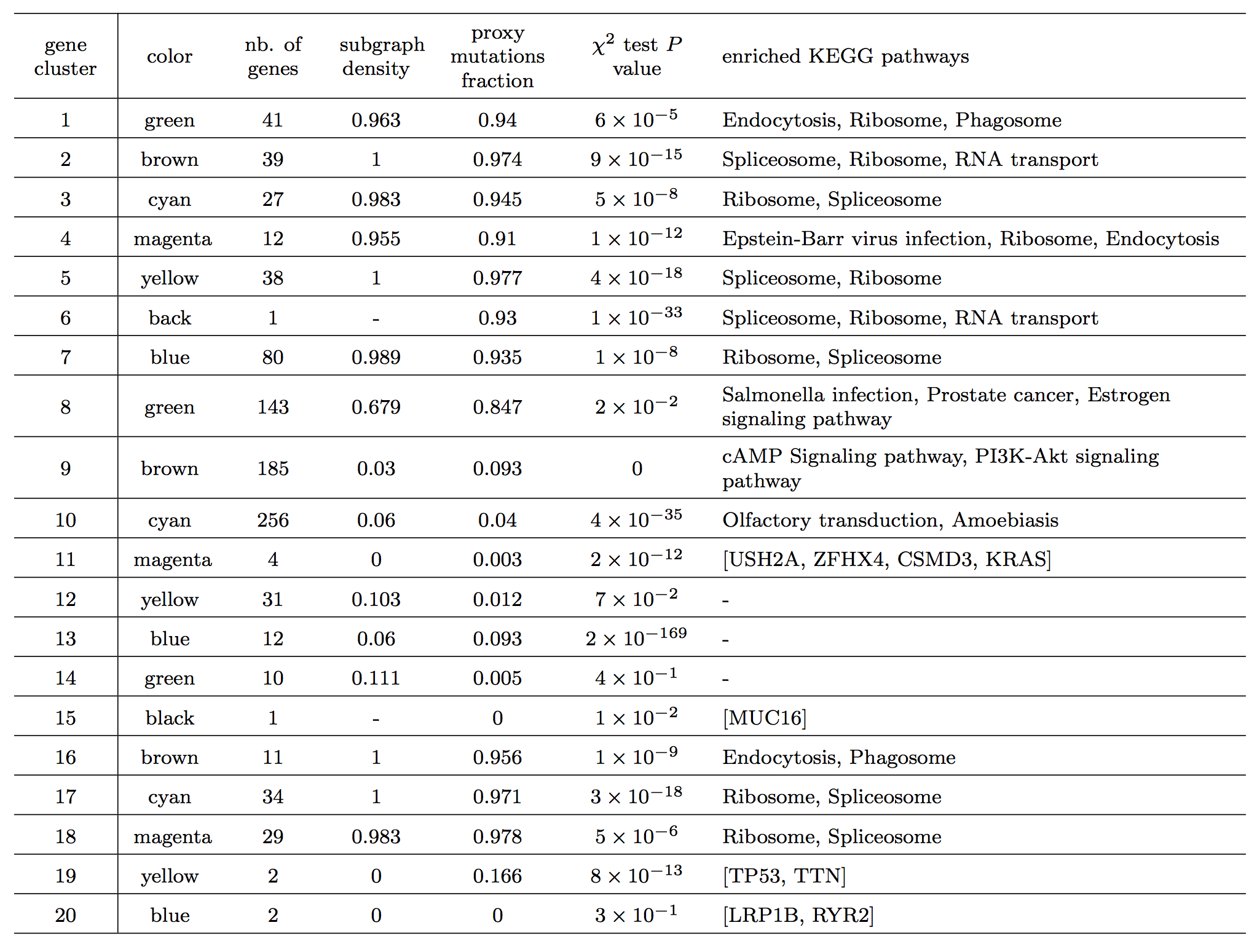

Supplement: S4 Table — nb. of genes: number of genes in a cluster, subgraph density: density of the subgraph whose vertices are the genes inside a cluster, proxy mutations fraction: number of proxy mutations out the the total number of mutations for a gene cluster across all patients. (TIFF) [file pcbi.1005573.s010.tiff]
